# Novel *Escherichia coli* active site *dnaE* alleles with altered base and sugar selectivity

**DOI:** 10.1111/mmi.14779

**Published:** 2021-07-31

**Authors:** Alexandra Vaisman, Krystian Łazowski, Martin A. M. Reijns, Erin Walsh, John P. McDonald, Kristiniana C. Moreno, Dominic R. Quiros, Marlen Schmidt, Harald Kranz, Wei Yang, Karolina Makiela‐Dzbenska, Roger Woodgate

**Affiliations:** ^1^ Laboratory of Genomic Integrity, National Institute of Child Health and Human Development National Institutes of Health Bethesda MD USA; ^2^ Laboratory of DNA Replication and Genome Stability, Institute of Biochemistry and Biophysics Polish Academy of Sciences Warsaw Poland; ^3^ MRC Human Genetics Unit, Institute of Genetics and Cancer The University of Edinburgh, Western General Hospital Edinburgh UK; ^4^ Gen‐H Genetic Engineering Heidelberg GmbH Heidelberg Germany; ^5^ Laboratory of Molecular Biology, National Institute of Diabetes and Digestive and Kidney Diseases National Institutes of Health Bethesda MD USA

**Keywords:** mutagenesis, replicase, replication fidelity, ribonucleotide excision repair, ribonucleotide incorporation, steric gate

## Abstract

The *Escherichia coli dnaE* gene encodes the α‐catalytic subunit (pol IIIα) of DNA polymerase III, the cell’s main replicase. Like all high‐fidelity DNA polymerases, pol III possesses stringent base and sugar discrimination. The latter is mediated by a so‐called “steric gate” residue in the active site of the polymerase that physically clashes with the 2′‐OH of an incoming ribonucleotide. Our structural modeling data suggest that H760 is the steric gate residue in *E.coli* pol IIIα. To understand how H760 and the adjacent S759 residue help maintain genome stability, we generated DNA fragments in which the codons for H760 or S759 were systematically changed to the other nineteen naturally occurring amino acids and attempted to clone them into a plasmid expressing pol III core (α‐θ‐ε subunits). Of the possible 38 mutants, only nine were successfully sub‐cloned: three with substitutions at H760 and 6 with substitutions at S759. Three of the plasmid‐encoded alleles, S759C, S759N, and S759T, exhibited mild to moderate mutator activity and were moved onto the chromosome for further characterization. These studies revealed altered phenotypes regarding deoxyribonucleotide base selectivity and ribonucleotide discrimination. We believe that these are the first *dnaE* mutants with such phenotypes to be reported in the literature.

## Graphical abstract

The accurate replication of every living organisms’ genome is achieved by high fidelity DNA polymerases. The enzymes not only need to choose the nucleotide with the correctly paired base (G, A, T or C), but also the right sugar (deoxyribonucleotide, not ribonucleotide). We report here, the construction and characterization of three novel active site mutants of the α‐catalytic subunit of *Escherichia coli* DNA polymerase III that have altered phenotypes with regard to base and sugar discrimination.

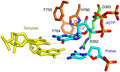

**Abbreviations**
*E. coli*
: *Escherichia coli*
pol: DNA polymerase
RER: ribonucleotide excision repair
Rif: rifampicin

## 1 INTRODUCTION

*Escherichia* *coli* (*E. coli*) possesses five DNA polymerases, among which pol III holoenzyme (pol III HE), a large asymmetric dimeric macromolecular complex, is the cell’s main replicase responsible for chromosome duplication by simultaneous coordinated leading and lagging strand synthesis (reviewed in Kornberg & Baker, 1992; Langston et al., 2009; McHenry, 2003, 2011; O’Donnell, 2006; Pomerantz & O’Donnell, 2007; Yao & O’Donnell, 2008). Pol III HE consists of 17 subunits [(αθε)_2_τ_2_γ_1_δδ′χψ(β_2_)_2_] encoded by nine genes expressing the α, β, ε, θ, δ, δ′, γ, τ, χ, and ψ polypeptides. The 130 kDa α‐subunit polymerase belongs to the C‐family of DNA polymerases and is encoded by the *dnaE* gene. The α‐subunit is usually found in a tight complex with the 27.5 kDa 3′→5′ proofreading exonuclease ε, encoded by the *dnaQ* gene; and the 8.6 kDa subunit θ, encoded by *holE*, which helps stabilize the three‐subunit pol III core sub‐assembly (Kim & McHenry, 1996; Kornberg & Baker, 1992).

Highly processive pol III HE synthesizes over 48 kb per binding event (Georgescu et al., 2011; Yao & O’Donnell, 2009) and replicates DNA with high speed (up to 1,000 nucleotides per second) (McInerney et al., 2007; Mok & Marians, 1987). As a result, under optimal growth conditions a single holoenzyme is sufficient to complete duplication of the entire 4 Mb *E. coli* genome in ~60 min (Fossum et al., 2007; McInerney & O’Donnell, 2004; Reyes‐Lamothe et al., 2010).

The fidelity of pol III has been extensively investigated *in vivo*. An important approach that significantly improved our understanding of the molecular mechanisms ensuring accurate replication of the bacterial chromosome is based on genetic selection and screening strains for altered fidelity (reviewed in [Fijalkowska et al., 2012]). Using this approach, several attempts were made to isolate mutations in the *dnaE* gene (Fijalkowska et al., 1993; Fijalkowska & Schaaper, 1993; Hiratsuka & Reha‐Krantz, 2000; Maki et al., 1991; Makiela‐Dzbenska et al., 2019; Oller et al., 1993; Oller & Schaaper, 1994; Sevastopoulos & Glaser, 1977; Sugaya et al., 2002; Vandewiele et al., 2002; Yanagihara et al., 2007), as well as genes encoding other pol III HE subunits (Fijalkowska & Schaaper, 1996; Gawel et al., 2008, 2011; Oller et al., 1993; Pham et al., 2006; Schaaper, 1993, 1996, 1998; Taft‐Benz & Schaaper, 1998, 2004) that conferred either antimutator or mutator phenotypes.

The isolation of a series of *dnaE* mutants that result in an altered replication fidelity uncovered an important role of wild‐type pol III in contributing to the normally low replication error rates in *E*. *coli* (Fijalkowska et al., 1993; Fijalkowska & Schaaper, 1993; Oller & Schaaper, 1994; Schaaper, 1993, 1996, 1998). Mapping of the various mutations, which are spread throughout the entire *dnaE* gene, suggested that although some of them are potentially located close to the active site of the enzyme (Kim et al., 1997; Lamers et al., 2006; Parasuram et al., 2018; Pritchard & McHenry, 1999), the effect on replication fidelity was more often indirect. One of the causes of an altered error rate can be a change in a variety of protein–protein interactions within the holoenzyme. For example, the significant *dnaE* mutator activity of the *dnaE173* (E612K) variant results from the reduced ability of the α‐subunit to interact with the ε‐proofreading subunit, thus disrupting coordination of the extension step mediated by the polymerase with the reverse proofreading step mediated by the exonuclease (Maki et al., 1990, 1991; Mo et al., 1991).

Besides generating base mispairs, DNA polymerases from all kingdoms of life often make mistakes by misincorporating ribonucleotides rather than deoxyribonucleotides. Indeed, due to the considerably greater intracellular concentration of rNTPs compared to dNTPs (up to 1,000‐fold in *E. coli*) (Bennett et al., 2009) ribonucleotides are inserted into DNA at substantial levels. *In vitro* experiments using physiological dNTP and rNTP concentrations show that pol III holoenzyme may incorporate up to 1 rNMP every 2.3 kb (Yao et al., 2013), and *in vivo*, the total number of rNMPs per *E. coli* genome has been estimated to be between 190 and 600 (Cronan et al., 2019; Kouzminova et al., 2017; Zatopek et al., 2019), in the absence of RNase HII (encoded by *rnhB*) a key enzyme in ribonucleotide excision repair (RER). Even though rNTPs and dNTPs have the same base‐coding potential, ribonucleotide incorporation might affect cellular mutability. This can occur due to the direct changes in polymerase fidelity, either during selection of a nucleotide substrate (rNTP vs. dNTP) (Brown & Suo, 2011; Donigan et al., 2014; Joyce, 1997), or during replication past rNMPs embedded in the template DNA strand (Donigan et al., 2014). In addition, errantly incorporated rNMPs appear to slow the replisome (Yao et al., 2013), which also might affect replication fidelity. Changes in cellular mutability due to rNTP incorporation can also be indirect and caused by the induction of RER. Such an effect has been shown recently in our studies with low‐fidelity *E. coli* pol V. Under certain conditions, wild‐type pol V promotes considerable levels of spontaneous mutagenesis. However, to our initial surprise, a pol V variant with decreased sugar selectivity resulted in a significant reduction of pol V‐dependent mutagenesis. We discovered that this is due to rNMP repair pathways triggered by misincorporated ribonucleotides. The main repair pathway for RER is initiated by RNase HII and completed by high‐fidelity pol I‐dependent nick translation that simultaneously removes rNMPs but also the pol V‐dependent misincorporated dNMPs, effectively resulting in an antimutator effect (Vaisman et al., 2013).

The major mechanism protecting cells from ribonucleotide incorporation is provided by DNA polymerases themselves. In most polymerases, ribose discrimination is determined by a single, so‐called “steric gate” residue that not only limits rNTP misincorporation, but also, given its location within the active site, can concurrently influence base selection and overall fidelity (Brown & Suo, 2011; Donigan et al., 2014; Sassa et al., 2019; Vaisman & Woodgate, 2018) as well as catalytic activity (DeLucia et al., 2006). Several studies have also shown that a significant role in rNTP discrimination might also be played by the residue immediately upstream of the steric gate, which controls base substitution fidelity (Brown & Suo, 2011; Nick McElhinny, Kumar, et al., 2010; Nick McElhinny, Watts, et al., 2010; Vaisman et al., 2012; Vaisman & Woodgate, 2018).

We have previously utilized steric gate mutants of pol V to investigate the molecular mechanisms of RER in *E*. *coli*. These studies led to the unexpected discovery that Nucleotide Excision Repair (NER) participates in ribonucleotide removal (Vaisman et al., 2013). Pol V is a slow and distributive DNA polymerase; we therefore wanted to extend our studies to a more robust and processive polymerase. To do so, we attempted to generate mutations in the cell’s main replicase, pol III, at the putative steric gate residue, H760, or the adjacent S759 residue in the α‐catalytic subunit of the polymerase. Here, we present data describing the initial characterization of three S759 mutants with differential impact on phenotypes with regard to base and sugar selectivity. These are the first pol III mutants with such phenotypes and their characterization provides considerable insights into how *E*. *coli* normally avoids the catastrophic consequences of high levels of errant deoxyribonucleotide and ribonucleotide incorporation during normal DNA replication.

## 2 RESULTS

### 2.1 Identification of the steric gate residue in the α‐catalytic subunit of *E. coli* pol III

**FIGURE 1:**
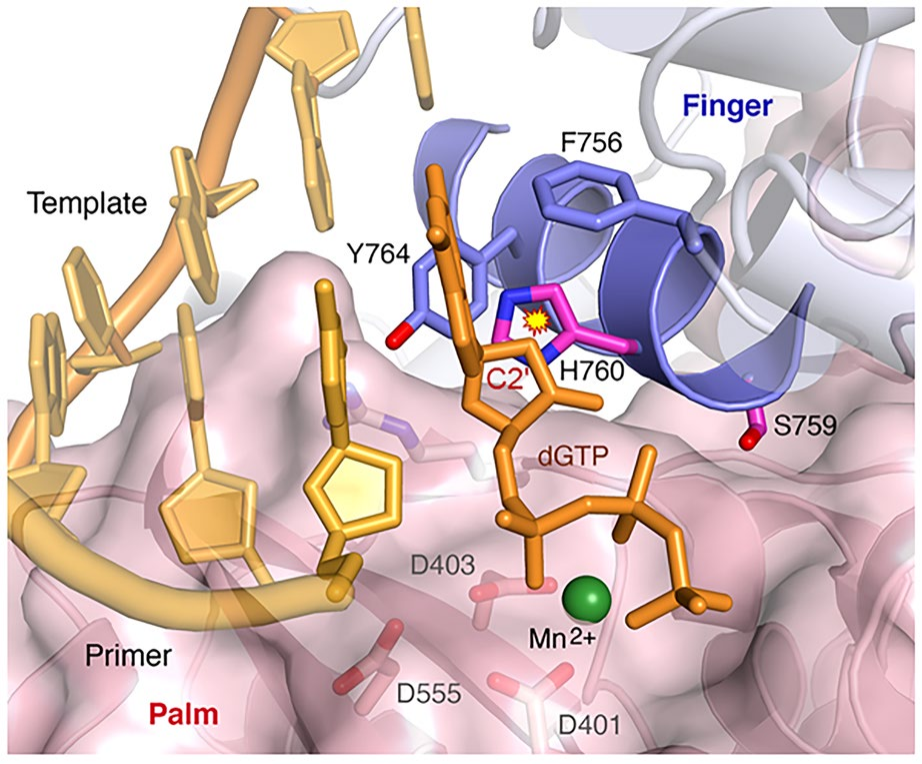
A model of the catalytic center of *Escherichia coli* DNA pol IIIα in a complex with DNA and dNTP substrate. The model was generated using the ternary complex structure of *Geobacillus kaustophilus* PolC (PDB: 3F2C (https://www.rcsb.org/structure/3F2C); Evans et al., 2008; See details described in Experimental Procedures). The secondary structures of the palm and finger domains that surround the catalytic center are shown in pink and blue, which are covered by a semi‐transparent molecular surface. The DNA template and primer strands are shown in gold with the last nucleotide in the primer strand and incoming dNTP shown as “sticks”. The active site residues D401, D403, and D555 (in the palm domain) are shown as pink sticks with red oxygen atoms. The α‐helix containing residues forming the steric gate (in the finger domain) is highlighted in dark blue, and the H760 and S759 residues analyzed in this manuscript are colored in magenta, while other key residues are colored blue. Dark blue and red colors in all stick models represent nitrogen and oxygen atoms, respectively. H760 directly contacts the deoxyribose of the incoming dNTP and forms the steric gate while S759 snuggly fits in a shallow pocket of the palm domain. The position of the 2′‐OH and its close proximity with H760 is marked by a collision sign (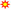)

At the time we initiated our studies, there were no high‐resolution ternary‐complex structures of the *E. coli* α‐catalytic subunit with DNA and dNTP substrate. The most detailed structural analysis was that of another C‐family polymerase, the α‐subunit encoded by *polC* from *Geobacillus kaustophilus* (*G. kaustophilus*) in a ternary complex with DNA and an incoming nucleotide (dGTP) at an atomic resolution of 2.4 Å (PDB ID codes 3F2B, 3F2C, and 3F2D; Evans et al., 2008). Based on this structure, we made a model of the active site of the α‐subunit of *E coli* pol III (pol IIIα; Figure 1) and concluded that the presumptive steric gate residue is H760. This amino acid is thought to be responsible for the prevention of the incorporation of nucleotides with a wrong sugar into DNA, due to the steric clash between the side chain of histidine with the 2′‐OH of an incoming ribonucleotide (Figure 1). Support for the idea that H760 plays an important role in sugar selectivity, came from a study by Parasuram et al., in which it was independently surmised that the H760 residue contributes to the recognition and interaction with the ribose moiety of the incoming nucleotide (Parasuram et al., 2018).

### 2.2 Generation of pol IIIα variants with amino acid substitutions at the steric gate residue H760, or the adjacent residue, S759

The α‐subunit replicates the genome in the context of the 17‐subunit pol III holoenzyme (Kornberg & Baker, 1992; McHenry, 2011; Pomerantz & O’Donnell, 2007), which can be fractionated into smaller complexes, including pol III core which comprises a tight sub‐assembly of α, ε, and θ subunits (Kim & McHenry, 1996). We therefore decided to make mutant variants of the α‐subunit that would be expressed in the context of pol III core. To do so, we generated pJM1260 (Table 1, and Figure  S1). This vector was designed with several expression and downstream purification options in mind. First, the genes encoding α, θ, and ε subunits (*dnaE, holE*, and *dnaQ*, respectively) were codon optimized for expression in *E*. *coli* and chemically synthesized (Genscript). The θ‐subunit was untagged, whilst ε was His‐tagged at its N‐terminus, and α was FLAG‐tagged at its N‐terminus for potential downstream affinity purification. To decrease the possibility that misincorporated bases would be subject to rapid proofreading by the ε‐subunit, we also introduced the *dnaQ920* (R56W) mutation into the *dnaQ* gene, which reduces the proofreading activity of the wild‐type ε‐subunit by ~90% (Taft‐Benz & Schaaper, 1998). The vector backbone for pJM1260 is based upon the low‐copy vector, pGB2 (~5‐copies per cell) (Churchward et al., 1984), so as to reduce any potential overproduction artifacts. However, the vector also contains the strong IPTG‐inducible pTrc promoter (de Boer et al., 1983) that could be used to induce the core complex for *in vivo* studies, or downstream purification.

**TABLE 1:** Plasmids used in this study

Plasmid	Relevant Characteristics	Source or reference
pJM1260	Low‐copy‐number plasmid expressing codon optimized pol III core (α, θ, ε)	This study
pJM1260‐*dnaE*_H760F	As pJM1260, but expressing *dnaE*_H760F	This study
pJM1260‐*dnaE*_H760Q	As pJM1260, but expressing *dnaE*_H760Q	This study
pJM1260‐d*naE*_H760S	As pJM1260, but expressing *dnaE*_H760S	This study
pJM1260‐*dnaE*_S759A	As pJM1260, but expressing *dnaE*_S759A	This study
pJM1260‐*dnaE*_S759C	As pJM1260, but expressing *dnaE*_S759C	This study
pJM1260‐*dnaE*_S759G	As pJM1260, but expressing *dnaE*_S759G	This study
pJM1260‐*dnaE*_S759N	As pJM1260, but expressing *dnaE*_S759N	This study
pJM1260‐*dnaE*_S759T	As pJM1260, but expressing *dnaE*_S759T	This study
pJM1260‐*dnaE*_S759V	As pJM1260, but expressing *dnaE*_S759V	This study
pALFIRE	Plasmid encoding for Red α/β and RecA expressed from the arabinose promoter and encoding the I‐*Sce*I restriction enzyme expressed from the anhydrotetracycline promoter	Rivero‐Müller et al. (2007)

The final α, ε, and θ expression vector, pJM1260, comprises 10,231 bp. Importantly, codons for S759 and H760 of the α‐subunit are located in a 453 bp region that is flanked by unique *Bst*BI and *Eag*I sites (Figure S1). We initially planned to synthesize 19 discrete *Bst*BI and *Eag*I fragments in which the steric gate H760 residue was changed to all 19 possible amino acids. However, we considered the possibility that some, or all, of these substitutions may be lethal, given the residue is invariant in all pol IIIα proteins. Therefore, we decided to also independently change the residue adjacent to the steric gate, S759. The precedent for such a change was based upon studies of steric gate mutants of *Saccharomyces cerevisiae* replicative DNA polymerases α, δ, and ε and *E. coli* DNA polymerase V, where changes to the residue adjacent to the steric gate led to altered base selectivity and sugar discrimination (Nick McElhinny, Kumar, et al., 2010; Nick McElhinny, Watts, et al., 2010; Vaisman et al., 2012).

Consequently, we synthesized thirty‐eight 453 bp DNA cassettes in which H760, or S759, were individually changed to the 19 other natural amino acids (Genscript). We then attempted to clone the DNA cassette into pJM1260 and were expecting a total of 38 variants. However, despite multiple attempts, we were only able to subclone three H760 variants (*dnaE_*H760F, *dnaE_*H760Q, *dnaE_*H760S) and six S759 variants (*dnaE_*S759A, *dnaE_*S759C, *dnaE_*S759G, *dnaE_*S759N, *dnaE_*S759T, *dnaE_*S759V) into DH5α (Table 1). We assume that our inability to subclone the remaining variants is due to “dominant negative” toxicity caused by the plasmid‐encoded mutant *dnaE* variant.

### 2.3 Functionality of plasmid encoded *dnaE* variants

The original plasmid clones were isolated from DH5α expressing chromosomal wild‐type α‐ and ε‐subunits. To determine if the plasmid‐encoded *dnaE* mutant alleles are functionally active, the respective plasmids were transformed into RW1138 lacking pol II, poI IV, and pol V (Table 2). In addition, this strain harbors the temperature sensitive *dnaE486* (S885P) allele (Wechsler & Gross, 1971) expressed from the chromosome, enabling it to grow on LB medium at permissive temperature (30℃), but not at non‐permissive temperatures (which in this DNA pol II‐, pol IV‐, and pol V‐deficient strain background is >37℃). As expected, wild‐type pol IIIα expressed from pJM1260 conferred temperature resistance to the normally temperature sensitive RW1138 strain (Table 3). Similarly, all six S759 alleles were able to confer temperature resistance to the RW1138 strain (Table 3). In contrast, *dnaE*_H760F, *dnaE*_H760Q, and *dnaE*_H760S failed to complement the temperature sensitivity of RW1138 (Table 3).

**TABLE 2:** *Escherichia coli* strains used in this study

Strain	Relevant genotype	Source or reference
P640	*dnaE*_S759T	Gen‐H
P648	*dnaE*_S759N	Gen‐H
P685	*dnaE*_S759C	Gen‐H
JW0198	*∆yafC727*::Kan	*E. coli* Genetic Stock Center
CAG18436	*∆yafC502*::*Tn10*	*E. coli* Genetic Stock Center
RW1606	*dnaE*_S759T ∆*yafC727*::Kan	P640 × P1. JW0198
RW1608	*dnaE*_S759N ∆*yafC727*::Kan	P648 × P1. JW0198
RW1712	*dnaE*_S759C ∆*yafC727*::Kan	P685 x P1. JW0198
RW1692	*dnaE*_S759N ∆*yafC502*::Tn*10*	P640 × P1. CAG18436
RW1720	*dnaE*_S759T ∆*yafC502*::Tn*10*	P648 × P1. CAG18436
RW1722	*dnaE*_S759C ∆*yafC502*::Tn*10*	P685 × P1. CAG18436
RW1138^a^	*dnaE486ts* ∆*yafC502*::Tn*10*	LGI^b^ stocks
RW1494^a^	∆*rnhB782*::Kan *dnaE486ts*	LGI stocks
RW1504^a^	*rnhB*_wt *dnaE486ts* ∆*yafC502*::Tn*10 dnaQ920*	LGI Stocks
RW1604^a^	∆*rnhB782 dnaE486ts* ∆*yafC502*::Tn*10 dnaQ920*	LGI Stocks
RW1726^a^	∆*rnhB782*::Kan *dnaE486ts dnaQ920*	LGI Stocks
RW1628^a^	*rnhB*_wt *dnaE_*wt *dnaQ*_wt	LGI Stocks
RW1610^a^	*rnhB*_wt *dnaE*_S759T ∆*yafC727*::Kan *dnaQ*_wt	RW1604 × P1. RW1606
RW1612^a^	*rnhB*_wt *dnaE*_S759N ∆*yafC727*::Kan *dnaQ*_wt	RW1604 × P1. RW1608
RW1714^a^	*rnhB*_wt *dnaE*_S759C ∆*yafC727*::Kan *dnaQ_wt*	RW1604 × P1. RW1712
RW1614^a^	*rnhB*_wt *dnaE*_wt ∆*yafC727*::Kan *dnaQ920*	RW1604 × P1. JW0198
RW1616^a^	*rnhB*_wt *dnaE*_S759T ∆*yafC727*::Kan *dnaQ920*	RW1604 × P1. RW1606
RW1618^a^	*rnhB*_wt *dnaE*_S759N ∆*yafC727*::Kan *dnaQ920*	RW1604 × P1. RW1608
RW1716^a^	*rnhB*_wt *dnaE*_S759C ∆*yafC727*::Kan *dnaQ920*	RW1604 × P1. RW1712
RW1630^a^	∆*rnhB782*::Kan *dnaE*_wt *dnaQ*_wt	LGI Stocks
RW1620^a^	∆*rnhB782 dnaE*_wt ∆*yafC727*::Kan *dnaQ920*	RW1604 × P1. JW0198
RW1624^a^	∆*rnhB782 dnaE*_S759T ∆*yafC727*::Kan *dnaQ*_wt	RW1604 × P1. RW1606
RW1626^a^	∆*rnhB782 dnaE*_S759T ∆*yafC727*::Kan *dnaQ920*	RW1604 × P1. RW1606
RW1718^a^	∆*rnhB782*::Kan *dnaE*_S759N ∆*yafC502*::Tn*10 dnaQ*_wt	RW1494 × P1. RW1692
RW1736^a^	∆*rnhB782*::Kan *dnaE*_S759C ∆*yafC502*::Tn*10 dnaQ*_wt	RW1726 × P1. RW1722
EC7344	*dnaQ920* ∆*yafC502*::Tn*10*	LDRGS^c^ stocks
EC10539^a^	*dnaQ920* ∆*yafC502*::Tn*10*	RW1628 × P1. EC7344
EC10540^a^	∆*rnhB782*::Kan *dnaE*_S759N	RW1628 × P1. RW1718
EC10541^a^	∆*rnhB782*::Kan *dnaE*_S759C	RW1628 × P1. RW1736
EC10544^a^	∆*rnhB782*::Kan *dnaE*_S759C ∆*yafC502*::Tn*10 dnaQ920*	EC10539 × P1. EC10541
EC10545^a^	∆*rnhB782*::Kan *dnaE*_S759N ∆*yafC502*::Tn*10 dnaQ920*	EC10539 × P1. EC10540

^a^ *thr‐1 Δ(argF‐lac)169 tsx‐33 supE44 galK2 hisG4* *rpsL31 xyl‐5 mtl‐1 argE3 thi‐1 sulA211 ∆(umuDC)596*::*ermGT ∆dinB61*::*ble* Δ*araD‐polB*::Ω.

^b^ Laboratory of Genomic Integrity

^c^ Laboratory of DNA Replication and Genome Stability

**TABLE 3:** Viability of strains expressing *dnaE* steric gate variants from pJM1260^a^

*dnaE* variant	10^−7^ CFU/ml	
	30℃	43℃
*dnaE*_wt	103 ± 7	107 ± 13
*dnaE*_H760F	234 ± 2	0 ± 0
*dnaE*_H760Q	314 ± 87	0 ± 0
*dnaE*_H760S	22 ± 13	0 ± 0
*dnaE*_S759A	83 ± 14	79 ± 19
*dnaE*_S759C	150 ± 34	158 ± 23
*dnaE*_S759G	149 ± 22	151 ± 20
*dnaE*_S759N	22 ± 4	18 ± 6
*dnaE*_S759T	41 ± 5	34 ± 5
*dnaE*_S759V	47 ± 20	40 ± 14

^a^ Viability assays were performed using *Escherichia coli* RW1138 (Table 2), which in the absence of a functional *dnaE* gene, grows at 30℃, but not at 37℃, or higher. CFU, colony forming unit. The values reported in the table are the average number of colonies obtained from three independent experiments (four plates each) ± standard error of the mean.

To assay whether the phenotypes of strains harboring the S759 or H760 plasmids corresponded to expression of the mutant pol IIIα subunits, we performed Western blot analysis of the mutant alleles expressed from pJM1260 (in the absence of IPTG induction). Extracts were probed with polyclonal rabbit antisera that we had previously raised to pol III core (unpublished results). This serum does not recognize the low chromosomal levels of the α‐subunit, but it does recognize the pJM1260 plasmid encoded wild‐type α‐subunit (but not ε‐ or θ‐subunits) with high specificity (Figure S2). In contrast, full‐length α‐subunit was not detected in extracts expressing H760F, H760Q, and H760S (unpublished observations). We conclude that *dnaE*_H760F, *dnaE*_H760Q, and *dnaE*_H760S plasmid encoded variants are highly unstable, in agreement with the observation that they were unable to complement the temperature sensitivity of the *dnaE486* allele *in vivo*. In contrast to the H760 variants, all the plasmid‐encoded S759 α‐subunit variants are readily detectible by Western blots producing signals of approximately similar intensities (Figure S2).

Since S759 is located in the active site of the α‐catalytic subunit, we anticipated that some pol IIIα variants might exhibit altered fidelity that would be manifested as a spontaneous mutator phenotype. To investigate this possibility, we introduced the plasmids into strain RW1504 (Table 2), which is similar to RW1138, but also carries the proofreading defective *dnaQ920* (R56W) allele on the chromosome and assayed for reversion of the *hisG4*(Oc) allele promoted by the mutant *dnaE* alleles at 30℃ (permissive temperature) or 39℃ (non‐permissive for the chromosomal *dnaE486* allele). The non‐permissive temperature in these experiments was lower than in the earlier studies (on LB medium) due to overall lower viability of all strains on the low‐histidine minimal medium used to monitor reversion of the *hisG4* allele.

**FIGURE 2:**
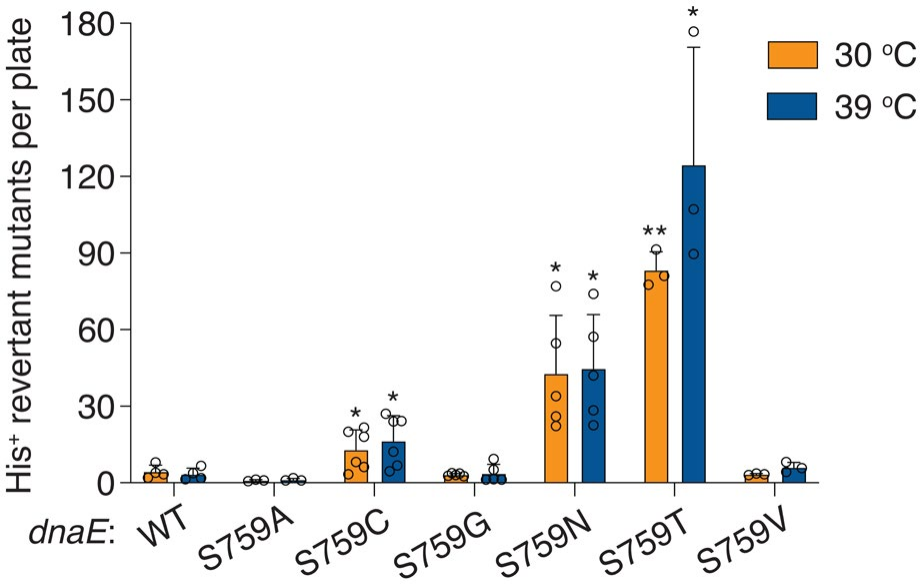
Quantitative His^+^ mutagenesis assays in RW1504 expressing S759 mutants. Strains were grown overnight at 30℃ in appropriate antibiotics. Aliquots were harvested by centrifugation and resuspended in an equal volume of SM buffer. 100 μl of the overnight culture was spread on each low‐histidine minimal plate and incubated at either 30℃ or 39℃, for four days, after which time, His^+^ revertants were counted. Symbols represent average counts for individual biological replicates (*n* = 3–6). Error bars represent one standard deviation. Unpaired, two‐tailed *t* tests were used to assess statistical significance between the mean colony counts for strains expressing wild‐type *dnaE* or *dnaE* variant, at 30℃ or 39℃. ^*^ *p* < .05, ^**^ *p* < .01. We did not detect a statistically significant difference in colony count between cultures grown at 30℃ or 39℃

The *dnaE*_S759A, *dnaE*_S759G and *dnaE*_S759V plasmid encoded *dnaE* variants exhibited low levels of spontaneous mutagenesis at both permissive and non‐permissive temperatures (Figure 2). In contrast, the *dnaE*_S759C, *dnaE*_S759N and *dnaE*_S759T alleles promoted progressively higher levels of spontaneous mutagenesis at both non‐permissive and permissive temperatures (Figure 2). Given that there is no indication of altered fidelity promoted by *dnaE*_S759A, *dnaE*_S759G, and *dnaE*_S759V, we chose not to characterize these alleles any further.

### 2.4 Moving the S759C, S759N, and S759T alleles onto the *E. coli* chromosome

To avoid any possible phenotypic artifacts promoted by the plasmid‐encoded FLAG‐tagged *dnaE* alleles expressed in the context of pol III core, we decided to move the untagged *dnaE*_S759C, *dnaE*_S759N or *dnaE*_S759T alleles onto the *E*. *coli* chromosome, where they would be expressed in the context of pol III holoenzyme. To do so, we employed Red/ET recombineering, as previously described by Kim et al. (2014), but with minor changes (see *Experimental procedures* and Table S1). During this process, the respective *dnaE* allele replaced the wild‐type *dnaE* gene. We then used conventional P1 transduction protocols to link the *dnaE* alleles to the nearby *yafC727*::Kan allele from JW0198 (*E*. *coli* Genetic Stock Center), or the *yafC502*::Tn*10* allele from CAG18436 (*E*. *coli* Genetic Stock Center). Previous studies have shown that *yafC* and *dnaE* are co‐transduced with a frequency of ~45% (Fijalkowska et al., 1993; Vandewiele et al., 2002; Figure S3). Finally, we transduced the respective *dnaE* alleles into the ∆*polB*, ∆*dinB*, and ∆*umuDC* strain, RW1604 (Table 2), so as to avoid any influence of other DNA polymerases on the replication fidelity and/or ribonucleotide incorporation. This strain also harbors the Δ*rnhB782*::Kan^S^ allele immediately upstream of *dnaE486ts* (Figure S3) and the *dnaQ920* (R56W) allele downstream of *yafC* (Figure S3). The *rnhB‐dnaE‐yafC‐dnaQ* interval is only ~33 kb in length, meaning that all four genes can be co‐transduced in a single P1 transduction (linkage of all four genes at one time is ~10%). Indeed, by screening for the appropriate gene marker, we were able to generate a series of *dnaE* strains that were deficient (Δ*rnhB*) or proficient (*rnhB*_wt) for RNase HII‐dependent‐RER, in the presence of fully active (*dnaQ*_wt) or reduced (*dnaQ920*) proofreading activity of pol III (Table 2).

Interestingly, while we were able to make wild‐type *dnaE* and *dnaE*_S759T strains that were Δ*rnhB dnaQ920* using selection for *yafC727*::Kan (Table 2), we were unable to make similar strains carrying the *dnaE*_S759C or *dnaE*_S759N alleles using this same approach. Instead, we used P1 lysates from strains in which ∆*rnhB782*::Kan was first linked to *dnaE*_S759C (EC10540), or *dnaE*_S759N (EC10541), to simultaneously transduce ∆*rnhB782*::Kan and the two S759C/N *dnaE* alleles into the ∆*yafC502*::Tn*10 dnaQ920* strain, EC10539 (Table 2).

### 2.5 Spontaneous mutagenesis promoted by chromosomally encoded *dnaE* variants

The effect of reduced proofreading activity on mutagenesis promoted by wild‐type *dnaE*, *dnaE*_S759C, *dnaE*_S759N, and *dnaE*_S759T was first investigated by qualitative plate assays that followed the reversion of the *hisG4*(Oc) (Figure S4) and *galK2*(Oc) alleles (Figure S5). These assays reveal that all three *dnaE* alleles expressed from the chromosome confer a mild spontaneous mutator phenotype. In the absence of proofreading, spontaneous mutator activity increased significantly, especially with the *dnaE*_S759N and *dnaE*_S759T alleles.

To more accurately determine effects of the three *dnaE* alleles on potential mutator activity, we used quantitative fluctuation assays to monitor forward mutagenesis to rifampicin resistance (mutations in *rpoB* gene encoding the β‐subunit of RNA polymerase). We compared the level of Rif mutagenesis promoted by wild‐type *dnaE* and the three *dnaE*_S759 variants in a repair‐proficient background, or in a background with altered RER (Δ*rnhB*), or proofreading activity (*dnaQ920*), or both (Δ*rnhB dnaQ920*; Table 4). In the repair‐proficient background, the *dnaE*_S759N allele displayed a moderate mutator effect, estimated to be ~7‐fold higher than wild‐type *dnaE*, while *dnaE*_S759T and *dnaE*_S759C alleles were lower mutators (3‐ and 1.7‐fold, respectively). Diminished proofreading (*dnaQ920*) in strains carrying *dnaE* alleles led to a further increase in mutagenesis, resulting in ~3.5–66‐fold mutator effects compared to the wild‐type *dnaE* ^+^ strain. Synergistic effects observed between *dnaE* variants and *dnaQ920* allele indicate that replication errors generated by all three mutants are subject to correction by the proofreading activity of pol III. Inactivation of the main RER pathway (Δ*rnhB*) in both proofreading proficient and proofreading deficient backgrounds had no significant effect on mutagenesis promoted by wild‐type *dnaE* or any of the three *dnaE* mutants (Table 4).

**TABLE 4:** Mutation rates of spontaneous rifampicin resistance in *dnaE* strains proficient, or deficient, in DnaQ and/or RNase HII activity

Genotype	Rif mutation rate × 10^9^			
	*dnaQ_*wt *rnhB_*wt	*dnaQ920 rnhB_*wt	*dnaQ_*wt Δ*rnhB*	*dnaQ920* Δ*rnhB*
*dnaE*_wt	2.04 (1.30−2.96)	12.8 (10.3−15.5)	1.49 (0.92−2.23)	11.0 (8.6−13.6)
*dnaE*_S759C	3.44 (2.86−4.08)	44.8 (39.8−49.7)	2.43 (1.66−3.36)	32.8 (26.5−39.2)
*dnaE*_S759N	14.0 (11.6−16.3)	854 (757−953)	12.4 (9.5−15.4)	805 (731−876)
*dnaE*_S759T	6.11 (4.55−7.81)	263 (222−303)	7.47 (5.70−9.38)	225 (187−266)

Note

Spontaneous *rpoB* mutation rates were measured in wild‐type, *dnaQ920*, and *ΔrnhB* genetic backgrounds. The mutation rates and 95% confidence intervals (in brackets) were calculated as described in Experimental Procedures (Zheng, 2017), using *n* = 15–57 cultures for each strain.

### 2.6 Different mutational spectra for *dnaE*_S759C, S759N, and S759T in proofreading deficient *dnaQ920* strains

We were interested in investigating the possibility that the *dnaE*_S759 variants might exhibit altered base substitution specificity in addition to their differing mutator phenotypes. To do so, we analyzed the mutation profiles of the mutant polymerases in a proofreading‐deficient (*dnaQ920*) background by determining the spectra of spontaneously arising missense mutations that lead to rifampin resistance. Such an approach has previously been utilized to show that each of *E*. *coli*’*s* five DNA polymerases exhibits a unique mutational signature (Curti et al., 2009; Garibyan et al., 2003; Makiela‐Dzbenska et al., 2011; Vaisman et al., 2013; Wolff et al., 2004). The strains used in this analysis are proficient for methyl‐directed mismatch repair (MMR), which is known to preferentially target transition mutations for repair (Schaaper & Dunn, 1987). As a consequence, we were expecting most of the mutations to be mismatch repair‐insensitive transversions. Indeed, wild‐type *dnaE* and *dnaE*_S759C have a high percentage of transversions, 90% and 93%, respectively (Table 5). In contrast, the *dnaE*_S759N and *dnaE*_S759T spectrum exhibited more transitions than transversions (~55% vs. ~45%). The increase in transition mutations with *dnaE*_S759N and *dnaE*_S759T alleles is likely due to the high levels of mutagenesis that overwhelm the mismatch repair machinery (Schaaper & Radman, 1989).

**TABLE 5:** Mutational changes in *rpoB* leading to rifampicin resistance of *Escherichia coli dnaQ920* strains expressing *dnaE_*wt and *dnaE_*S759 variants

bp change	*dnaE*_wt	*dnaE*_S759C	*dnaE*_S759N	*dnaE*_S759T
CG→GC	2 (0.7%)	5 (1.3%)	0 (0%)	0 (0%)
CG→AT	8 (2.6%)	15 (4.0%)	0 (0%)	5 (1.5%)
CG→TA	9 (3%)	12 (3.2%)	104 (31.8%)	106 (30.8%)
AT→TA	89 (29.5%)	316 (85.2%)	140 (42.8%)	152 (44.2%)
AT→CG	174 (57.6%)	10 (2.7%)	3 (0.9%)	1 (0.3%)
AT→GC	20 (6.6%)	13 (3.5%)	80 (24.5%)	80 (23.3%)
Transitions	29 (10.6%)	25 (6.7%)	184 (56.3%)	186 (54.1%)
Transversions	273 (90.4%)	346 (93.3%)	143 (43.7%)	158 (45.9%)
Total	302	371	327	344

Note

Data shown in brackets are number of particular base substitutions calculated as a percent of total mutations, or the number of transitions or transversions calculated as a percent of total mutations.

**FIGURE 3:**
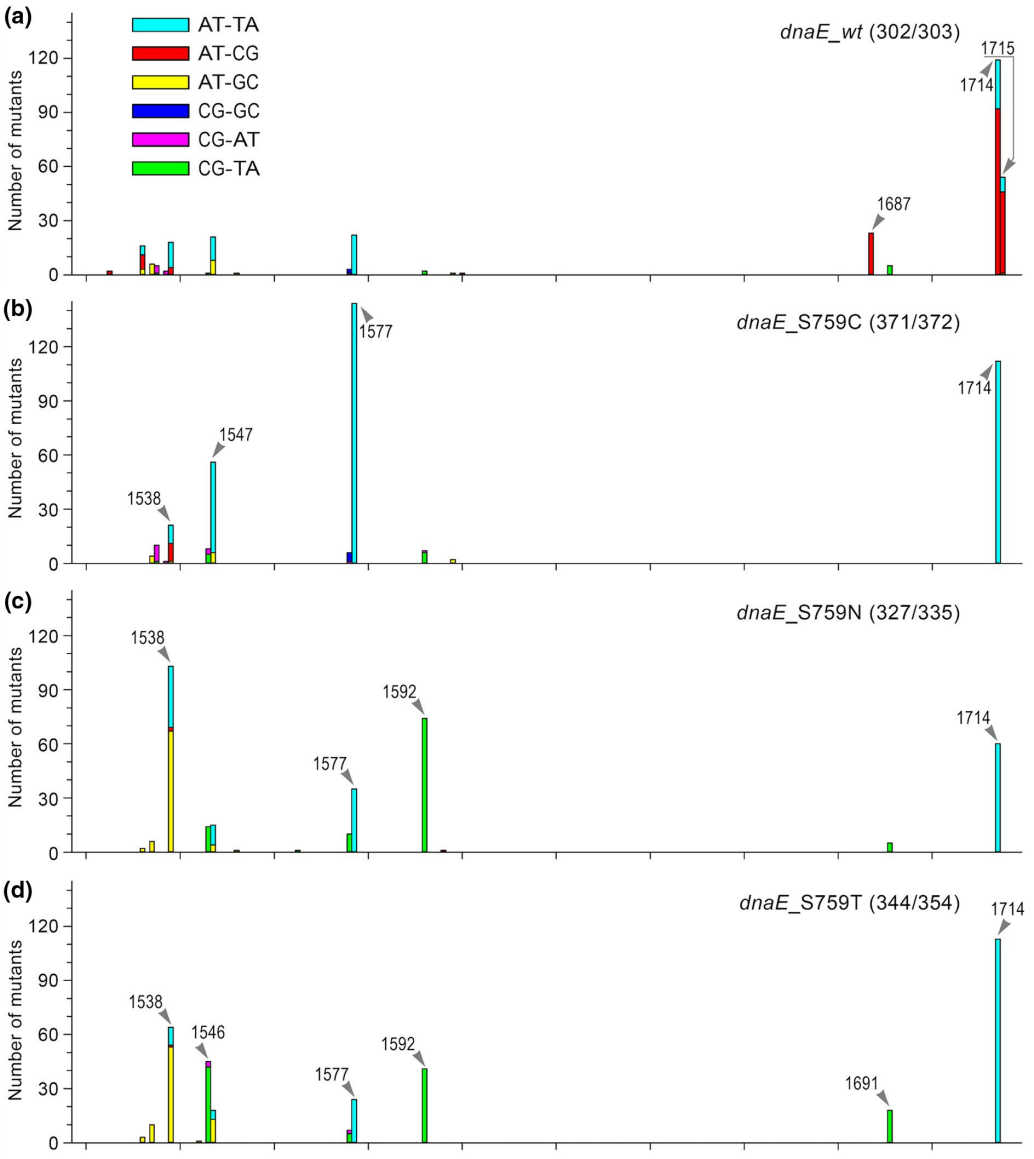
Spectra of spontaneous mutations in the *rpoB* locus in a *dnaQ920* proofreading‐deficient background. (a) Wild‐type *dnaE*, (b) *dnaE*_S759C, (c) *dnaE*_S759N, or (d) *dnaE*_S759T. The types of base‐pair substitutions observed in the *rpoB* gene that result in rifampicin resistance are color coded as shown in the figure. The arrows indicate mutagenic hot spots. The numbers in brackets next to the name of the *dnaE* allele refer to the number of mutants identified/number of mutants assayed. A more detailed spectral analysis can be found in Table S2

In addition to variability in transitions versus transversions, the pattern of base substitutions in the *rpoB* locus also differ between wild‐type *dnaE* and the three variants (Tables 5 and S2 and Figure 3). For example, the predominant mutation in wild‐type *dnaE* is AT→CG (~58%, mostly accumulated within hot spots at positions 1687, 1714, and 1715, Figure 3a), yet this mutagenic event comprises just 2.7%, 0.9%, and 0.3% in *dnaE*_S759C, *dnaE*_S759N, and *dnaE*_S759T strains, respectively (Figure 3b‐d). Even in the DNA site especially prone to undergo base changes in all strains tested (position 1714, Figure 3), the types of mutations recovered from wild‐type *dnaE* and the three variants were different. In the strain with wild‐type *dnaE*, the majority of mutations found at position 1714 were AT→CG transversions, while in other strains they were AT→TA substitutions (Figure 3). Other notable differences include a dramatic increase in the occurrence of AT→TA transversions in the *dnaE*_S759C strain (~85%, most prominent at three mutagenic peaks at positions 1547, 1577, and 1714, Figure 3b). The types of base changes and mutagenic hot‐spots in the *dnaE*_S759N and *dnaE*_S759T strains were very similar to each other (Table 5 and Figure 3c,d), with the exception of additional CG→TA transitions at positions 1546 and 1691 in the *dnaE*_S759T strain (Figure 3d). We speculate that differences in the types of base substitutions are directly due to the altered misincorporation specificity of the individual *dnaE* allele.

### 2.7 Increased ribonucleotide incorporation promoted by *dnaE*_S759C, *dnaE*_S759N, and *dnaE*_S759T

**FIGURE 4:**
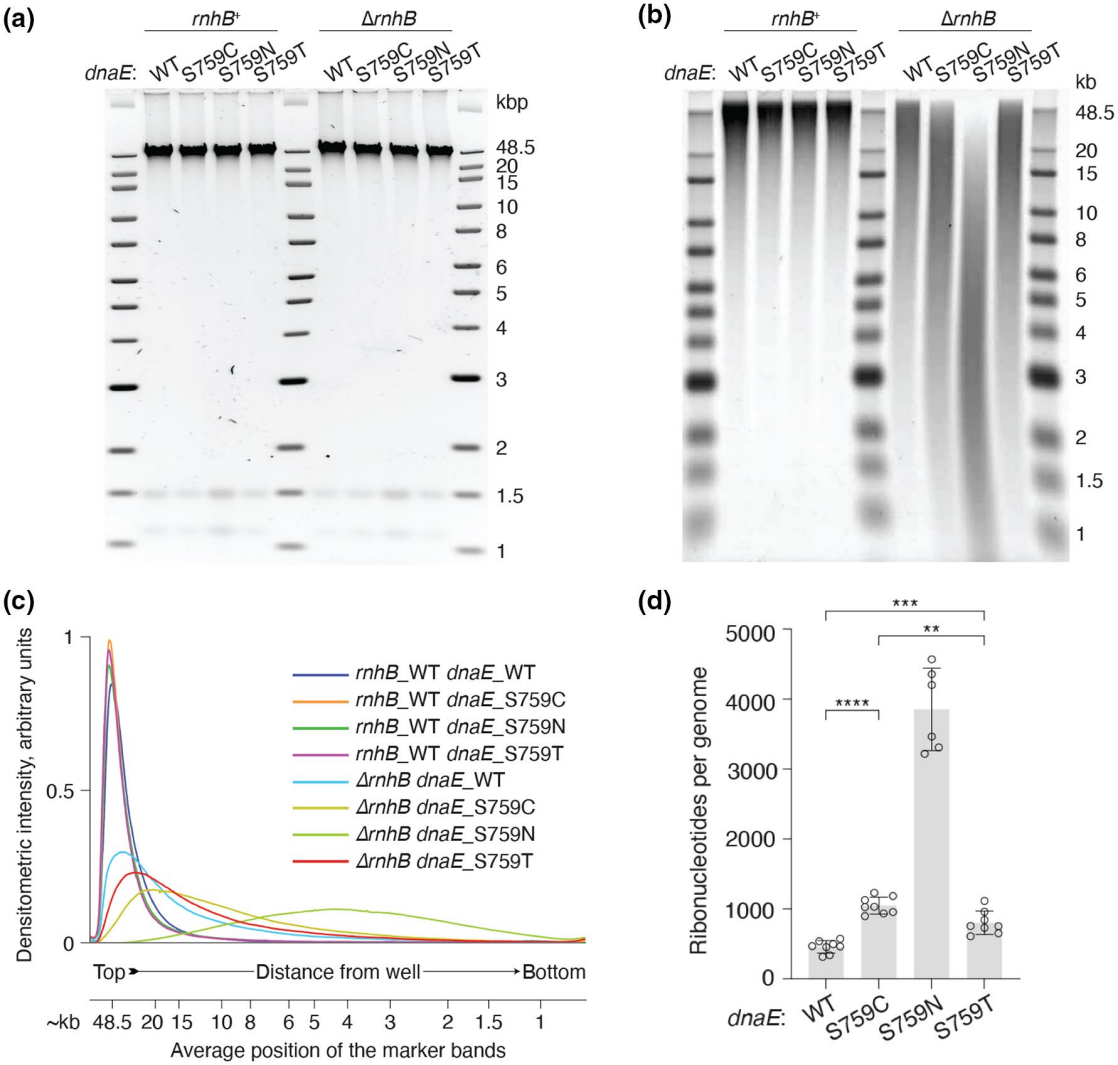
Increased ribonucleotide incorporation by *dnaE* steric gate mutants. (**a**) High molecular weight genomic DNA isolated from *rnhB*_wt and Δ*rnhB Escherichia coli* with wild‐type or *dnaE* variants separated by TAE agarose gel electrophoresis (b) RNase H2‐treated genomic DNA separated by alkaline gel electrophoresis (representative of ≥6 independent experiments). (c) Densitometric intensity plots for the gel shown in panel B show greater fragmentation in the Δ*rnhB* strains, indicating higher numbers of genome‐embedded ribonucleotides. (d) Densitometry plots were used to calculate the number of ribonucleotides per Δ*rnhB* genome relative to *rnhB*_wt strains, showing significantly increased levels in *dnaE*_S759T (1.8‐fold), *dnaE*_S759C (2.3‐fold), and *dnaE*_S759N strains (8.4‐fold) compared to *dnaE*_wt. Individual data points indicate values from *n* = 6–8 independent experiments, with bars and error bars indicating mean ± *SD*. Unpaired 2‐sided *t*‐test with Welch’s correction; **p < .01; ***p < .001; ****p < .0001

To determine the impact of the three S759 variants on ribonucleotide incorporation, we performed alkaline gel electrophoresis of RNase H2‐treated genomic DNA (Figure 4). No change in migration was observed for *rnhB*_wt strains due to efficient RER (Figures 4 and S6), whereas RER‐deficient Δ*rnhB* strains showed increased fragmentation, indicative of the presence of more genome‐embedded ribonucleotides, consistent with previous findings (Kouzminova et al., 2017; Zatopek et al., 2019). For Δ*rnhB* cells expressing the three *dnaE*_S759 variants the number of embedded ribonucleotides was further elevated. The smallest change in the fragmentation pattern was observed for *dnaE*_S759T; *dnaE*_S759C gave an intermediate effect, while the largest effect was observed for *dnaE*_S759N (Figures 4 and S6).

Using the densitometry measurements after alkaline gel electrophoresis, we calculated the frequency of embedded ribonucleotides in genomic DNA from *ΔrnhB E. coli* to be 49 ± 9.6 ribonucleotides per million bases (mean ± *SD*, *n* = 8 independent experiments) (Table 6), which is in line with previous experiments using alternative methods that estimated between 20 and 130 embedded ribonucleotides per million bases (Cronan et al., 2019; Kouzminova et al., 2017; Zatopek et al., 2019). Importantly, our analyses showed significantly increased ribonucleotide incorporation rates as a result of the *dnaE* S759T, S759C, and S759N mutations. We estimate the increase relative to wild‐type *dnaE* to be 1.8, 2.3, and 8.4‐fold respectively, with misincorporation in the *ΔrnhB dnaE*_S759N strain as high as one ribonucleotide every ~2.5 kb (Figure 4 and Table 6).

**TABLE 6:** Ribonucleotides (rN) embedded in the genome of Δ*rnhB* strains expressing wild‐type *dnaE* or *dnaE*_S759 variants^a^

Genotype	rN per genome^b^	rN per Mb	Kb per rN	Fold difference^c^	*p*‐value^d^
*dnaE*_wt	457 ± 89	49 ± 9.6	21.1 ± 4.7	1.00	1.00
*dnaE*_S759C	1,049 ± 122	113 ± 13	9.0 ± 1.1	2.30	<.0001
*dnaE*_S759N	3,854 ± 589	415 ± 63	2.5 ± 0.4	8.44	<.0001
*dnaE*_S759T	803 ± 167	86 ± 18	12.0 ± 2.3	1.76	.0003

^a^ Numbers shown: mean ± standard deviation of *n* = 6–8 independent measurements.

^b^ Based on an *Escherichia coli* genome of 4.64 Mbp (i.e., 9.28 Mb).

^c^ Difference between mean values for wild‐type *dnaE* and individual S759 variants.

^d^ *p*‐values calculated using unpaired 2‐sided *t*‐test with Welch’s correction for rN per genome relative to *dnaE*_wt.

## 3 DISCUSSION

The aim of the current study was to make a series of *E*. *coli* pol IIIα variants with amino acid substitutions at the presumptive steric gate residue, H760, and the adjacent residue, S759. Given their location in the active site of the enzyme, we anticipated that some would have effects on base and/or sugar selection during replication. We originally envisaged being able to construct 38 “active site” mutants in the pol IIIα subunit. However, after repeated cloning attempts, we were only able to generate nine new variants. We assume that our inability to make the remaining 29 possible variants is due to synthetic‐lethality of the strain when it is transformed with the plasmid‐encoded mutant. Three of the novel mutants were located at H760, but Western blots of the α‐subunit encoded *dnaE*_H760F, *dnaE*_H760Q, and *dnaE*_H760S indicate that they are highly unstable and/or poorly expressed (unpublished observations).

All six S759 variants expressed the α‐subunit at approximately similar levels and were able to complement the temperature sensitivity of the *dnaE486* allele. Three plasmid‐encoded variants, S759C, S759N, and S759T also exhibited modest to substantial increases in spontaneous mutagenesis *in vivo*. The alleles were moved to the *E. coli* chromosome to avoid any possible plasmid‐encoded phenotypic artifacts and subjected to a variety of *in vivo* assays to determine their ability to misincorporate nucleotides with an incorrect base or sugar.

The *E*. *coli* strains used in these studies lack DNA pol II, IV, and V, so any replication associated phenotypes can only be attributed to the remaining DNA polymerases: wild‐type pol I, or the different pol IIIα variants. We also chose to conduct our studies in a mismatch repair proficient background, so that we could study the effects of the α‐subunit S759 mutants on transition and transversion mutagenesis. The studies were enhanced through the comparison of phenotypes of strains with a wild‐type repair proficient background to Δ*rnhB* strains lacking RNase HII, which is essential for the majority of RER; and/or a *dnaQ920* strain, which is severely compromised for exonucleolytic 3′→5′ proofreading. Phenotypes in the Δ*rnhB* background were hypothesized to reflect differences in the ability of the *dnaE* variant to incorporate ribonucleotides into the *E. coli* genome, whereas phenotypes in the *dnaQ920* background would reflect differences in accurate/erroneous base selection.

With all three mutant alleles, reduction in the proofreading activity of pol III resulted in a synergistic increase in spontaneous mutagenesis, indicating that errors generated by the mutant polymerases are normally subject to ε‐dependent proofreading. Analysis of the genomic DNA fragmentation pattern based on ribonucleotide‐induced alkali sensitivity in the Δ*rnhB* background revealed that *dnaE*_S759N and to a lesser extent *dnaE*_S759C and *dnaE*_S759T have reduced ribonucleotide discrimination (Figure 4). However, inactivation of RER by the Δ*rnhB* allele had a minimal effect on levels of spontaneous mutagenesis promoted by the three *dnaE* alleles. Such phenotypes are likely to be expected for the *dnaE*_S759T or *dnaE*_S759C alleles that exhibit limited ribonucleotide incorporation. However, the fact that there was no difference in the levels of spontaneous mutagenesis in the Δ*rnhB dnaE*_S750N strain implies that RER is unlikely to concomitantly remove dNTPs misincorporated by *dnaE*_S759N.

Based upon the data presented here, we suggest that the three *dnaE* mutants have differential phenotypes regarding base and sugar selection.

*dnaE*_S759C: This variant exhibited a low spontaneous mutator activity, even in a *dnaQ920* background (Table 4). We therefore conclude that the *dnaE*_S759C variant maintains a high degree of base selectivity. In contrast, analysis of ribonucleotide incorporation (Figure 4 and Table 6), indicates that it incorporates 2.3‐fold higher levels of ribonucleotides compared to wild‐type *dnaE*, indicating that sugar discrimination in this variant is at least partially compromised.

*dnaE*_S759T: This variant exhibited a low spontaneous mutator activity in a proofreading proficient (*dnaQ*_wt) background, but a high mutator phenotype in a *dnaQ920* background (Table 4, Figures  S4 and S5). This indicates that base errors generated by *dnaE*_S759T are normally efficiently proofread *in vivo*. Furthermore, the spectrum of *rpoB* mutations was unlike that of wild‐type *dnaE* (Figure 3) and exhibited a substantial increase in transition mutations (Table 5). We therefore conclude that the *dnaE*_S759T variant has much lower base fidelity than wild‐type *dnaE*. Similar to *dnaE*_S759C, ribonucleotide incorporation (Figure 4 and Table 6), is elevated ~1.8‐fold compared to wild‐type *dnaE*, indicating that in addition to very low base selectivity, sugar discrimination is partially compromised.

*dnaE*_S759N: This variant exhibited spontaneous mutator activity that was similar to *dnaE*_S759T (Table 4, Figure S4 and S5). However, analysis of ribonucleotide incorporation promoted by *dnaE*_S759N indicates that sugar selectivity is severely compromised (Figure 4 and Table 6). As a result, the *dnaE*_S759N mutant incorporates ribonucleotides at an ~8.4‐fold higher rate than wild‐type *dnaE*, which equates to the incorporation of an errant ribonucleotide every ~2.5 kb in the *E*. *coli* genome. Thus, the *dnaE*_S759N allele is compromised for both base and sugar discrimination.

### 3.1 Structural basis for the observed phenotypes of *dnaE*_S759C, *dnaE*_S759N, and *dnaE*_S759T

The three *dnaE*_S759 alleles are adjacent to the steric gate that we have identified as H760 (Figure 1). We assume that the various phenotypes observed *in vivo* are due to direct changes in the ability of the α‐subunit to misincorporate dNTPs and/or rNTPs. The H760 residue is in the “O” helix of the polymerase in the finger domain, both of which undergo conformational changes from an “open” state in the absence of an incoming dNTP to a “closed” state when the polymerase is ready to incorporate a dNTP (Doublie et al., 1999; Evans et al., 2008). S759 butts against the polymerase Palm domain. When S759 is changed to Cys, Thr, or Asn, it causes steric clashes with the Palm domain, which contains the catalytic triad and metal ion (Figure 1). The most likely scenario is that the O helix does not close properly on the replicating base pair, thus loosening base selection and/or sugar discrimination.

### 3.2 Future considerations

To the best of our knowledge, this is the first time that active site mutants of the α‐catalytic subunit of pol III characterized by differential phenotypes regarding base and sugar discrimination as well as different mutational specificity have been reported. We believe that these novel mutants, due to their versatility, provide us with new tools that open new possibilities to study how *E*. *coli* normally maintains high fidelity replication and avoids the deleterious consequences of ribonucleotide misincorporation. Such studies will also need to be accompanied by the determination of the structures of appropriate enzyme–substrate complexes, which should provide an explanation of how modification of the active site architecture affects the substrate specificities and characteristics of each polymerase variant. We also plan to carry out detailed biochemical analysis of the purified α‐subunits *in vitro* in the context of α‐alone, pol III core and pol III holoenzyme, so as to elucidate novel features of the structural and molecular mechanisms that give rise to the differential phenotypes of the S759 mutants *in vivo*. Last, but not least, we hope that the pol III variants which are characterized by different base and sugar fidelities will help us to determine whether prokaryotic cells employ the same set of repair pathways for cleansing genomic DNA of ribonucleotides incorporated by replicative and translesion DNA polymerases.

## 4 EXPERIMENTAL PROCEDURES

### 4.1 Bacterial strains and plasmids

Plasmids used in this study are described in Table 1.

Most of the *E. coli* K‐12 strains used in this study are derivatives of RW732 (full genotype: *thr‐1 araD139* ∆(*gpt‐proA*)*62 lacY1 tsx‐33 supE44 galK2 hisG4 rpsL31 xyl‐5 mtl‐1 argE3 thi‐1 sulA211 ΔumuDC596*::*ermGT ΔaraD‐polB*::*Ω ΔdinB61*::*ble*) (Table 2). All derivatives of RW732 were made by standard methods of P1 transduction using P1*vir* (Table 2).

Where noted, bacteria were grown on LB agar plates containing 20 μg/ml chloramphenicol; 15 μg/ml tetracycline; 25 μg/ml zeocin; 50 μg/ml kanamycin; 20 μg/ml spectinomycin; or 100 μg/ml rifampicin.

### 4.2 Construction of a low‐copy number plasmid expressing pol III core

The genes encoding the α, ε, and θ subunits (*dnaE, dnaQ*, and *holE*, respectively) were codon optimized for expression in *E. coli* (Genscript) and synthesized as gene cassettes with appropriate 5′ and 3′ restriction enzyme sites for subsequent subcloning. The starting vector was the low copy number ampicillin vector pJM975 (Frank et al., 2012). The Lac repressible‐IPTG‐inducible pTrc promoter (de Boer et al., 1983) was first cloned into pJM975 as a 184 bp *Eco*RI‐*Nde*I fragment. Next, the *dnaQ920* gene with an R56W substitution in *dnaQ* was cloned into this vector as a 790 bp *Nde*I‐*Bam*HI fragment, to generate pJM1048, which expresses N‐terminal His‐tagged DnaQ920. *holE* and the 5′ end of the FLAG‐tagged *dnaE* gene was subsequently subcloned into pJM1048 as a 2,540 bp *Bam*HI‐*Bpu*10I fragment. The 3′ end of the *dnaE* gene was then subcloned as a 1,351 bp *Bpu*10I‐*Xho*I fragment, so as to reconstruct the full‐length *dnaE* gene and generate the pol III core destination vector, pJM1260 (Figure S1).

### 4.3 Use of a temperature sensitive *dnaE486* strain to determine if plasmid encoded *dnaE*s are functionally active

To determine if the plasmid encoded *dnaE* mutant alleles are functionally active, plasmid DNAs were transformed into RW1138, which harbors the temperature sensitive *dnaE486* allele (S885P) and grown at permissive temperature of 30℃. Transformants were then grown in liquid culture at 30℃ overnight. The following morning, serial dilutions of the individual cultures were made and plated on LB agar plates at permissive (30℃) and non‐permissive (43℃) temperatures. Plasmid encoded *dnaE* alleles were deemed to be fully functional if equal numbers of viable colonies were obtained at both permissive and non‐permissive temperatures.

### 4.4 Western blots

RW1138 (*dnaE486*ts) (Table 2) harboring pJM1260 (wild‐type *dnaE*), or S759 *dnaE* variants (Table 1) were grown overnight at 30℃ in LB medium plus appropriate antibiotics. The next morning, cultures were diluted 1∶100 in fresh LB and grown with aeration at 30℃ until they reached an OD_600_ of ∼0.5. Cells were then; (i) centrifuged; (ii) resuspended in 1× NuPAGE™ LDS sample buffer (Invitrogen, NP0007; 106 mM Tris·HCl, 141 mM Tris base, 2% LDS, 10% glycerol, 0.51 mM EDTA, 0.22 mM SERVA blue G250, 0.175 mM phenol red, pH 8.5) containing 2% β‐mercaptoethanol; (iii) immediately frozen in dry ice; (iv) lysed by multiple freeze‐thaw cycles; and (v) heated for 10 min at 70℃. Extracts were applied to a 4%–12% NuPAGE Bis‐Tris gel (Invitrogen, NP0321). After separation, proteins were transferred to an Invitrolon PVDF membrane (Invitrogen, LC2005) using standard Western blot protocols. The membrane was incubated overnight with rabbit polyclonal antibodies (1∶1,000 dilution) raised against the α‐θ‐ε subunits of pol III core (Covance, PA). The membrane was then incubated with secondary goat anti‐rabbit alkaline phosphatase conjugated antibodies (1:10,000 dilution) (BioRad, 1706518) and visualized using the Tropix CDP‐Star assay (Applied Biosystems, T2306). Images were captured on a FluorChem HD2 imaging system (ProteinSimple).

### 4.5 His^+^ and Gal^+^ reversion assays

#### 4.5.1 Quantitative assays

To assay the effect of plasmid encoded *dnaE* variants on spontaneous mutagenesis, RW1504 was freshly transformed with one of the pJM1260 *dnaE*‐variant plasmids described in Table 1 and grown overnight at 30℃ on LB plates containing ampicillin. Five well‐separated colonies were picked and inoculated into 5 ml LB/ampicillin medium and grown overnight with shaking at 30℃. The next day, cultures were harvested by centrifugation and resuspended in an equal volume of SM medium (Sambrook et al., 1989). Aliquots (100 μl) of each culture were plated in triplicate on Davis and Mingioli minimal plates (Davis & Mingioli, 1950) (1% agar, 0.4% glucose, 0.25 μg/ml thiamine, 0.7% potassium hydrogen orthophosphate, 0.2% potassium dihydrogen orthophosphate, 0.1% ammonium sulfate, 0.25% trisodium citrate, and 0.01% magnesium sulfate) supplemented with 100 μg/ml of L‐arginine, L‐valine, L‐leucine, L‐threonine, L‐leucine, L‐proline, and 1 μg/ml L‐histidine. Plates were incubated at 30℃, or 39℃ for 4 days, after which time His^+^ revertant colonies were counted. The data shown are the mean number of His^+^ revertants data obtained from five individual colonies plated in triplicate for each strain.

#### 4.5.2 Qualitative assays

To assay the effect of chromosomally encoded *dnaE* variants on spontaneous mutagenesis, the *dnaE* strain was grown overnight at 37℃ in LB medium containing the appropriate antibiotics. For His^+^ reversion assays, the cultures were processed as described above. For Gal^+^ reversion assays, overnight cultures were serial diluted in SM medium and ~50 to 100 bacteria plated on MacConkey agar base plates containing 1% galactose. Plates were incubated at 37℃ for 8 days before checking for the appearance of Gal^+^ (red) papillae arising from the predominantly Gal^−^ (pink/orange) colony.

### 4.6 Moving *dnaE_S759* alleles to the *E*. *coli* chromosome

The introduction of the *dnaE*_S759C, *dnaE*_S759N, and *dnaE*_S759T alleles into the essential *dnaE* gene of *E. coli* MG1655 was performed according to Kim et al, with minor changes (Kim et al., 2014). In a first recombineering step, a linear mutation cassette was introduced via Red/ET recombination using the plasmid pALFIRE (Rivero‐Müller et al., 2007) into the chromosomal *dnaE* gene resulting in a hybrid *dnaE* gene (*dnaE*_hybrid_ in Figure S7) encoding for a fully functional DNA polymerase III α‐subunit. The mutation cassette consists of (i) a 50 to 100 bp homology arm corresponding to the wild‐type *dnaE* gene (*dnaE*_wt_ in Figure S7), (ii) a fragment encoding an alternative nucleotide sequence of the *dnaE* gene (*dnaE*_alt_ in Figure S7) containing the desired point mutation at position 759, (iii) an I‐*Sce*I restriction site, (iv) a chloramphenicol selection marker (*cat* in Figure S7), (v) a second fragment of the wild‐type *dnaE* gene (Δ*dnaE*_wt_) again containing the desired point mutation, followed by a second 50 to 100 bp long homology arm corresponding to the wild‐type *dnaE* gene. In a second step, the selection marker was removed via RecA mediated repair using a I‐*Sce*I restriction site as the selection strategy as described by Rivero‐Müller et al. (2007). Finally, all clones were analyzed by DNA sequencing the modified region.

### 4.7 Moving chromosomal *dnaE* alleles into *rnhB* ^+^/Δ*rnhB* and *dnaQ* ^+^/*dnaQ920* strains

Once the respective *dnaE* allele had been successfully moved to the chromosome of the wild‐type *E. coli* strain, MG1655, we used conventional P1 transduction protocols to transfer the alleles into various repair‐deficient genetic backgrounds (Table 2). To do so, we first linked the respective *dnaE* allele to the nearby *yafC727*::Kan allele from JW0198 (*E. coli* Genetic Stock Center), or the *yafC502*::Tn*10* allele from CAG18436 (*E. coli* Genetic Stock Center). Depending upon the genotype of the recipient strain and existing antibiotic resistance, transductants were either selected for resistance to kanamycin, or tetracycline, and then screened for co‐transduction of the respective *dnaE* allele by colony PCR (see below), and/or phenotypic traits, such as conferring temperature resistance to the temperature sensitive *dnaE486ts* parental strain, or a spontaneous mutator phenotype (See Figures S4 and S5). Due to their close genomic location (Figure S3), *yafC* also co‐transduces with *rnhB* and *dnaQ* with high efficiency, and transductants were also screened by colony PCR to ascertain the status of the *rnhB* (*rnhB*_wt vs. Δ*rnhB*) and *dnaQ* (*dnaQ*_wt vs. *dnaQ920*) genes.

### 4.8 Colony PCR assay to test for *rnhB*, *dnaE*, and *dnaQ* alleles

A sterile pipette tip was used to pick a small quantity of bacteria from the purified P1 transductants and were then subject to PCR amplification. The primers used to amplify *rnhB* were rnhB_F‐55 and rnhB_R773 (Table S1). PCR amplification was achieved by denaturation at 95℃ for 5 min, followed by 60 cycles of 94℃ for 30 s, 1 min at 59℃, 2 min at 72℃, followed by a final extension step at 72℃ for 7 min. rnhB_F‐55 and rnhB_R773 amplifies 711 bp of intact *rnhB*, 977 bp of Δ*rnhB*::Kan, or 204 bp of Δ*rnhB*::Kan^S^.

The primers used to amplify *dnaE486* were EcdnaE486_F2378 and EcdnaE486_R2911 (Table S1). Amplification was achieved by denaturation at 95℃ for 5 min, followed by 30 cycles of 94℃ for 30 s, 1 min at 55℃, 2 min at 72℃, followed by a final extension step at 72℃ for 7 min. The primers amplify a 571 bp region surrounding *dnaE486* (S885 [TCC] → P885 [CCC]). *dnaE486* is cut with *Sma*I/*Xma*I into 293 and 278 bp fragments. The primers used to amplify *dna*E_S759 alleles were dnaE_F2059 and dnaE_R2557 (Table S1). Amplification was achieved by denaturation at 95℃ for 5 min, followed by 60 cycles of 94℃ for 30 s, 1 min at 57℃, 2 min at 72℃, followed by a final extension step at 72℃ for 7 min. dnaE_F2059 and dnaE_R2557 amplifies 537 bp surrounding the S759 codon. S759T and S759C both create a new *Bsl*I site. Digestion with *Bsl*I of S759T and S759C PCR amplicons gives a 225 bp fragment and a 299 bp fragment. The *dnaE*_S759N allele does not change a restriction site, so it was confirmed by DNA sequencing.

The primers used to amplify *dnaQ920* were EcdnaQ_F26 and EcdnaQ_R328 (Table S1). Amplification was achieved by denaturation at 95℃ for 5 min, followed by 60 cycles of 94℃ for 30 s, 1 min at 57℃, 1.5 min at 72℃, followed by a final extension step at 72℃ for 7 min. The primers amplify a 341 bp amplicon which gives fragments of 156 bp and 185 bp after digestion with *Pvu*I. *dnaQ920* (R56 [CGG] →W56 [TGG]) destroys the *Pvu*I site.

### 4.9 Spectra of spontaneous mutations in *rpoB*

The mutation spectra were generated using the *rpoB* mutagenesis assay (Garibyan et al., 2003). A single pair of oligonucleotide primers were used for PCR amplification and a single primer for DNA sequencing because 88% of all *rpoB* mutations are localized in the central 202 bp region of the gene (Garibyan et al., 2003). *E. coli* strains were diluted from a frozen stock cultures so that the initial inoculum contained <1,000 viable cells. For spectral analysis of *rpoB* mutants, several hundred independent LB cultures were grown for 24 hr at 37℃ in parallel for each strain, and appropriate dilutions were plated on an LB agar plate containing 100 µg/ml rifampicin. Using a pipette tip one colony was picked randomly from each plate to ensure independence of the mutants. About 400 independent Rif resistant colonies were obtained for each strain and subjected to PCR in a 96‐well micro‐titer plate. An ~1 kb central region of the *rpoB* gene was amplified using the PCR primers RpoB1 and RpoF1 (Table S1) by denaturation at 95℃ for 3 min, followed by 30 cycles of 94℃ for 30 s, 1 min at 59℃, 2 min at 72℃, followed by a final extension step at 72℃ for 7 min. The ∼200 bp target region of *rpoB* in each PCR amplicon was sequenced by TACGen (Richmond, CA) Genomics using WOG923AP01 primer (5′‐CAG TTC CGC GTT GGC CTG‐3′). Only base‐pair substitutions occurring between positions 1,516 and 1,717 of the *rpoB* gene were considered during data analysis. Nucleotide sequences obtained were aligned and analyzed using the ClustalW multiple sequence alignment program (Hinxton, UK).

### 4.10 Fluctuation assay for determination of forward mutation rates

For the fluctuation analysis, 15–57 cultures were inoculated with single colonies and grown overnight (~18 hr) at 37℃. Aliquots (100 μl) of each overnight culture, undiluted or diluted (10‐fold), were plated on agar plates containing 100 μg/ml of rifampicin and incubated for 24–36 hr at 37℃. To determine the colony forming units (CFU), 50 μl of appropriate dilutions of the same cultures were plated on LB plates and incubated for 18–24 hr at 37℃. Mutation rates were calculated using the Maximum Likelihood Estimate (MLE) method (Rosche & Foster, 2000; Sarkar et al., 1992) with a Newton‐Raphson‐type algorithm modified to account for partial plating, available in a free R package rSalvador (Zheng, 2015, 2017). This calculator also computes 95% confidence intervals and employs Likelihood Ratio Test to calculate the statistical significance of the differences between mutation rates of various strains (Zheng, 2016). To account for multiple comparisons, the *p* values were adjusted using the Benjamini‐Hochberg procedure (Benjamini & Hochberg, 1995).

### 4.11 Genomic DNA isolation

Genomic DNA used for alkaline gel electrophoresis was isolated from *rnhB*_wt strains; RW1628 (*dnaE*_wild‐type), RW1714 (*dnaE*_S759C), RW1612 (*dnaE*_S759N), and RW1610 (*dnaE*_S759T) and Δ*rnhB* strains; RW1630 (*dnaE*_wild‐type), RW1736 (*dnaE*_S759C), RW1718 (*dnaE*_S759N), and RW1624 (*dnaE*_S759T; Table 2), using a previously described method (Ding et al., 2015). In brief, *E. coli* from 1.5 ml of overnight culture was pelleted and resuspended in 200 μl of lysis buffer (2% Triton X‐100, 1% SDS, 0.5 M NaCl, 10 mM Tris, and 1 mM EDTA, pH 8.0). Cells were lysed by vortexing in the presence of 0.2 ml glass beads (0.4–0.6 mm diameter) and 200 μl of phenol (pH 7.9) for 2 min, then another min after adding 200 μl of TE buffer. After subsequent extractions with 400 μl of phenol:chloroform:isoamylalcohol (25:24:1) and 400 μl of chloroform, DNA was precipitated with ice‐cold ethanol. DNA was quantified using the Qubit dsDNA BR Assay (Invitrogen), and quantity and quality checked by agarose gel electrophoresis (0.8%, 1× TAE).

### 4.12 Alkaline gel electrophoresis

Genomic DNA was treated with RNase H2 and separated by alkaline gel electrophoresis, essentially as described (Benitez‐Guijarro et al., 2018). In brief, genomic DNA (250 ng) was treated with 1 pmol of purified recombinant human RNase H2 (Reijns et al., 2011) and 0.25 μg of DNase‐free RNase (Roche) for 1 hr at 37℃ in 100 μl reaction buffer (60 mM KCl, 50 mM Tris–HCl pH 8.0, 10 mM MgCl_2_, 0.01%Triton X‐100). Nucleic acids were ethanol precipitated and separated by alkaline gel electrophoresis (0.7% agarose, 50 mM NaOH, 1 mM EDTA). After electrophoresis, the gel was neutralized in 0.7 M Tris–HCl pH 8.0, 1.5 M NaCl and stained with SYBR Gold (Invitrogen). Images were taken using the FLA‐5100 imaging system (Fujifilm), and densitometry plots generated using AIDA Image Analyzer (Raytest).

### 4.13 Quantification of genome‐embedded ribonucleotides

Numeric analysis was performed in R (version 3.5.2) and Microsoft Excel 2016. Plotting and statistical tests were carried out in GraphPad Prism (version 9.1.1). The number of genome‐embedded ribonucleotides was estimated using a combination of previously described methods (Reijns et al., 2012; Uehara et al., 2018). Starting from the densitometric histograms per lane after alkaline gel electrophoresis, background intensity was uniformly subtracted and smoothed by fitting the smooth.spline function with 40 degrees of freedom in R. Peaks in the molecular weight marker lanes (NEB Quick‐Load 1 kb Extend DNA ladder) were identified under supervision and the linear model lm(*y*~log(*x*)) fitted to produce electrophoretic distance (*y*; mean of all marker lanes per gel) to fragment size (*x*) calibration curves. The resulting model was then applied to calculate the fragment size (sz) per electrophoresis distance interval, and fragment count per interval (*n*_sz_) estimated as *n*_sz_ = *I*_sz_/sz, with *I*_sz_ the densitometric intensity for the interval. To avoid fragment count errors resulting from noise near the bottom of the alkaline gel, where small changes in staining intensity would result in relatively large changes in the inferred number of small fragments, a cut‐off electrophoretic distance (*d*_max_) was introduced, with the corresponding molecular weight as the minimum fragment size (sz_min_). The sum of n_sz_ in all intervals (Σ*n*_sz_) down to *d*_max_ and the sum across the same intervals for sz·*n*_sz_ (Σ[sz·*n*_sz_]) were then used to determine a preliminary estimate of mean fragment size for each sample: s̅z̅ = Σ(sz·*n*_sz_)/ Σ*n*_sz_, and a correction applied for small fragments migrated beyond *d*_max_, to give a corrected mean fragment size s̅z̅_corr_ = s̅z̅ · exp(−sz_min_/s̅z̅). For a genome of size G (9.28 × 10^6^ nt for MG1655) the number of breakpoints for each sample was then calculated as N = G/s̅z̅_corr_. Additional break points (i.e., an estimate of the number of embedded ribonucleotides per genome) in *ΔrnhB* DNA were computed as N_ribo_ = N_Δ*rnhB*_−N_WT_. As there was no significant difference in the corrected mean fragment sizes for the different *rnhB* ^+^ strains, N_WT_ was taken as the mean of N for all *rnhB* ^+^ samples per gel. To determine the statistical significance of differences in s̅z̅_corr_ or N_ribo_ between samples across 6–8 independent experiments, an unpaired 2‐sided *t*‐test with Welch’s correction was performed.

### 4.14 Molecular modeling

Although structures of *E. coli* pol IIIα in the apo and DNA‐complex form have been reported (Fernandez‐Leiro et al., 2015; Lamers et al., 2006), a ternary‐complex structure with DNA and incoming dNTP is not available yet. The ternary complex structure of the C‐family DNA polymerase from *G. kaustophilus* (PDB: 3F2C; Evans et al., 2008) offers the best resolution (2.5 Å) view of the catalytic center engaging in DNA synthesis and is a relevant model for *E. coli* pol IIIα because of the conserved amino acid sequence in the region (24% identity and 39% similarity). Indeed, the apo structure of *E. coli* DNA pol IIIα (PDB: 2HQA (https://www.rcsb.org/structure/2HQA); Lamers et al., 2006) was superimposable with the catalytic core of *G*. *kaustophilus* PolC, which includes residues 825 to 1,102 encompassing the palm and thumb domains. The structure superposition confirms the sequence alignment, and a model of the *E. coli* DNA pol IIIα ternary complex was thus generated as shown in Figure 1.

## CONFLICT OF INTEREST

The authors declare that they have no conflict of interest with the content of this article.

## AUTHOR CONTRIBUTIONS

*Conceptualization*: J.P. McDonald, R. Woodgate; *Data Curation*: A. Vaisman, M.A.M. Reijns, K. Łazowski, K. Makiela‐Dzbenska; *Formal Analysis*: A. Vaisman, E. Walsh, M.A.M. Reijns, K. Łazowski, K. Makiela‐Dzbenska: *Funding Acquisition*: K. Makiela‐Dzbenska, K. Łazowski, W. Yang, R. Woodgate: *Investigation*: A. Vaisman, K. Łazowski, M.A.M. Reijns, E. Walsh, J.P. McDonald, K.C. Moreno, D.R. Quiros, M. Schmidt, W. Yang, K. Makiela‐Dzbenska, R. Woodgate; *Visualization*: A. Vaisman, E. Walsh, M.A.M. Reijns, K. Łazowski, K. Makiela‐Dzbenska, W. Yang; *Writing‐Original Draft*: A. Vaisman, E. Walsh, J.P. McDonald, M.A.M. Reijns, W. Yang, K. Makiela‐Dzbenska, R. Woodgate; *Writing‐Review and Editing*: A. Vaisman, K. Łazowski, M.A.M. Reijns, E. Walsh, J.P. McDonald, K.C. Moreno, D.R. Quiros, M. Schmidt, H. Kranz, W. Yang, K. Makiela‐Dzbenska, R. Woodgate.

## Supporting information

Supplementary Material

## Data Availability

The data that support the findings of this study are available from the corresponding author upon reasonable request.
