# Supplementary material for: Novel Escherichia coli active site dnaE alleles with altered base and sugar selectivity
Source: Mol Microbiol. 2021 Jul 31;116(3):909–25. doi: 10.1111/mmi.14779 (PMC8485763; doi:10.1111/mmi.14779)
Supplement: Supplementary file 1 — Supplementary Material [file MMI-116-909-s001.pdf]

molecular microbiology

Supplementary Information

Novel *Escherichia coli* active site *dnaE* alleles with altered base and sugar selectivity

Alexandra Vaisman, Krystian Łazowski, Martin A. M. Reijns, Erin Walsh, John P. McDonald, Kristiniana C. Moreno, Dominic R. Quiros, Marlen Schmidt, Harald Kranz, Wei Yang, Karolina Makiela-Dzbenska and Roger Woodgate

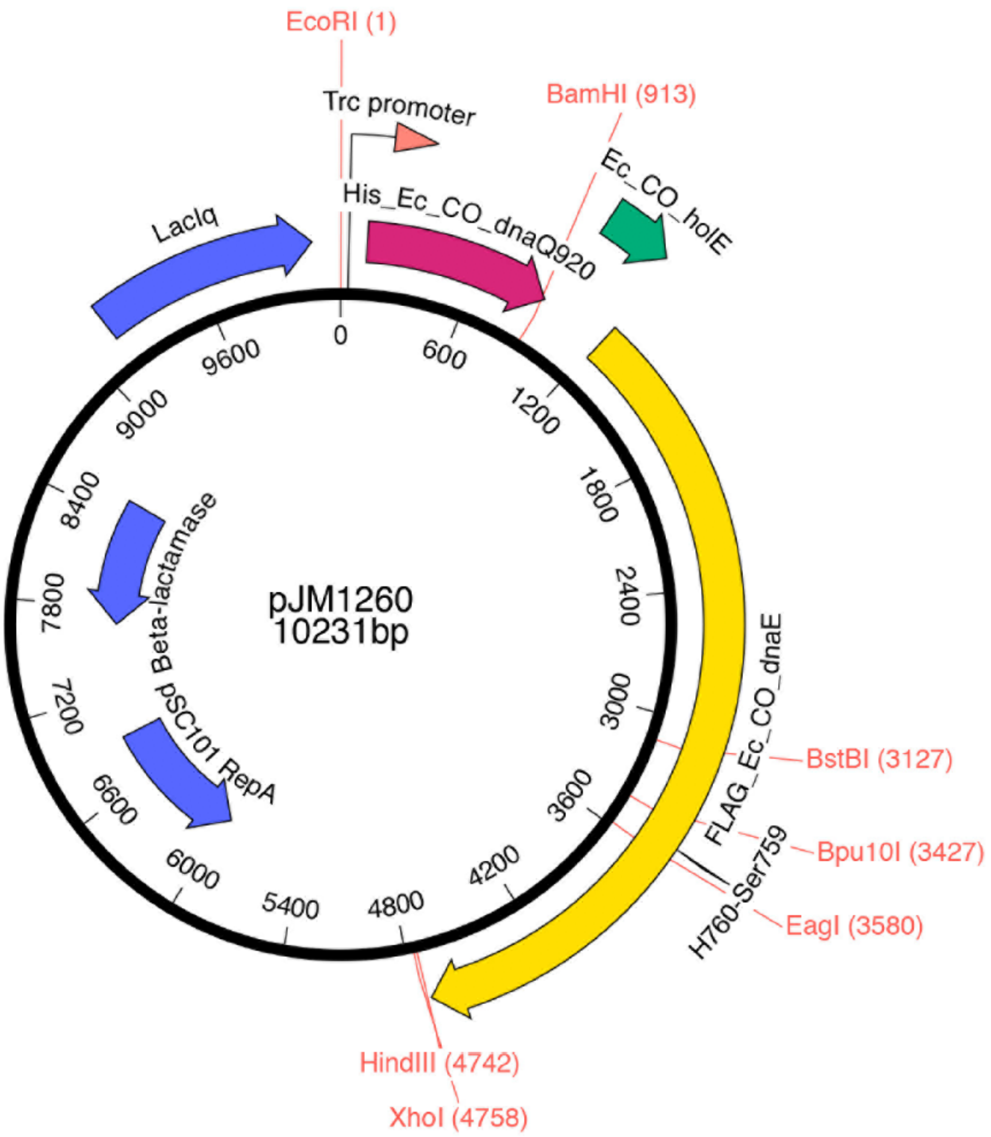

Figure S1. Cartoon of the pol III core vector, pJM1260. This 10,231 bp low-copy number plasmid expresses N-terminal HIS-tagged ε, untagged θ and N-terminal FLAG-tagged α. Steric gate substitutions can be generated by subcloning a chemically synthesized DNA fragment containing the desired substitution into the unique *Bst*BI and *Eag*I restriction enzyme sites.

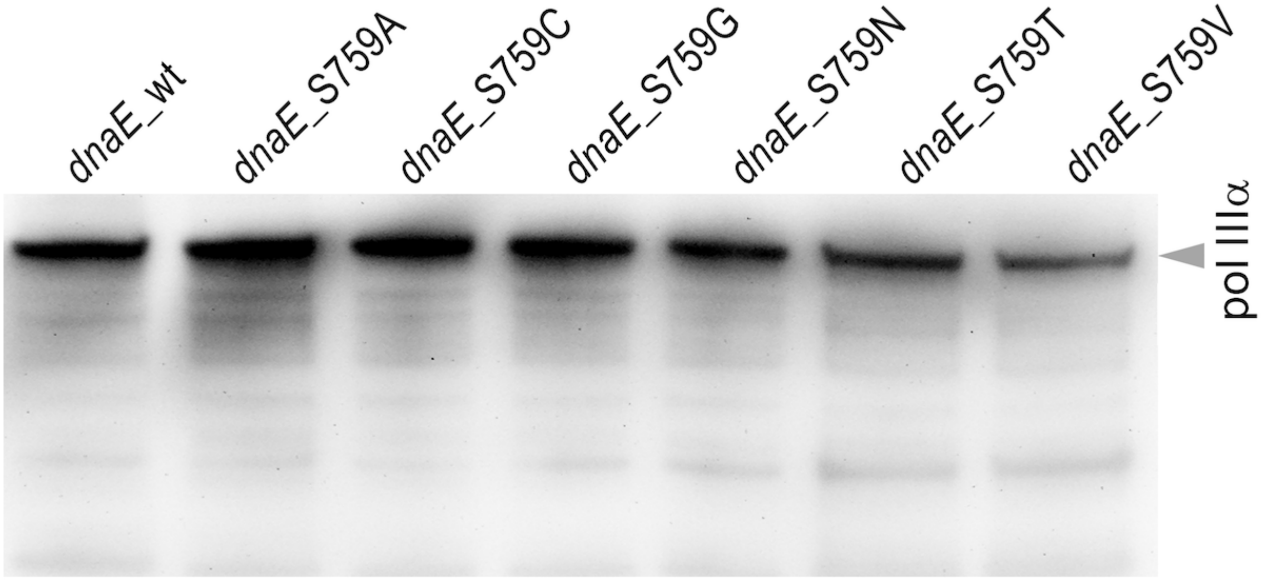

Figure S2. Western blot of the plasmid encoded α-subunit of pol III core expressed from pJM1260 and derivatives. The α-subunit of pol III core expressed from plasmid pJM1260 and variants were detected in whole cell extracts from *E. coli* RW1138 using polyclonal rabbit antibodies raised against pol III core. The position of full-length pol IIIα is indicated on the right-hand side of the image.

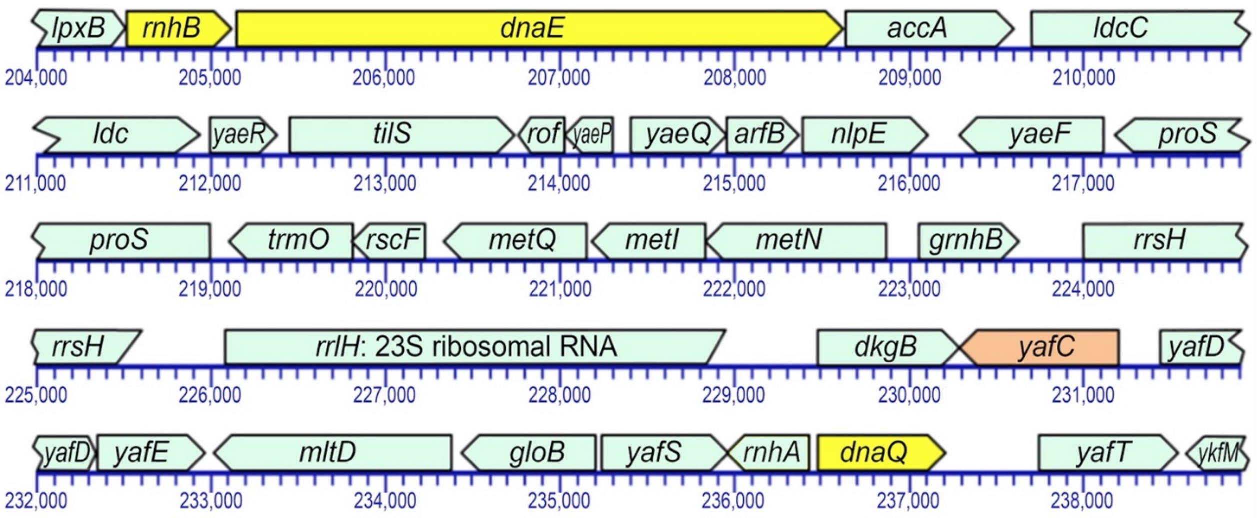

Figure S3. Schematic structure of the *lpxB-yklM* interval of the *E. coli* chromosome. The position of *yafC*, which was marked with kanamycin, or tetracycline resistance, for selection purposes, is shown in pale orange. The position of *dnaE, dnaQ, and rnhB* genes are highlighted in yellow. Co-transduction of *yafC* with *dnaE* was estimated to be ~50%; linkage to *dnaQ920* was ~44%; and to *rnhB* it was ~32%. All four genes can be co-transduced at one time with a frequency of ~10%.

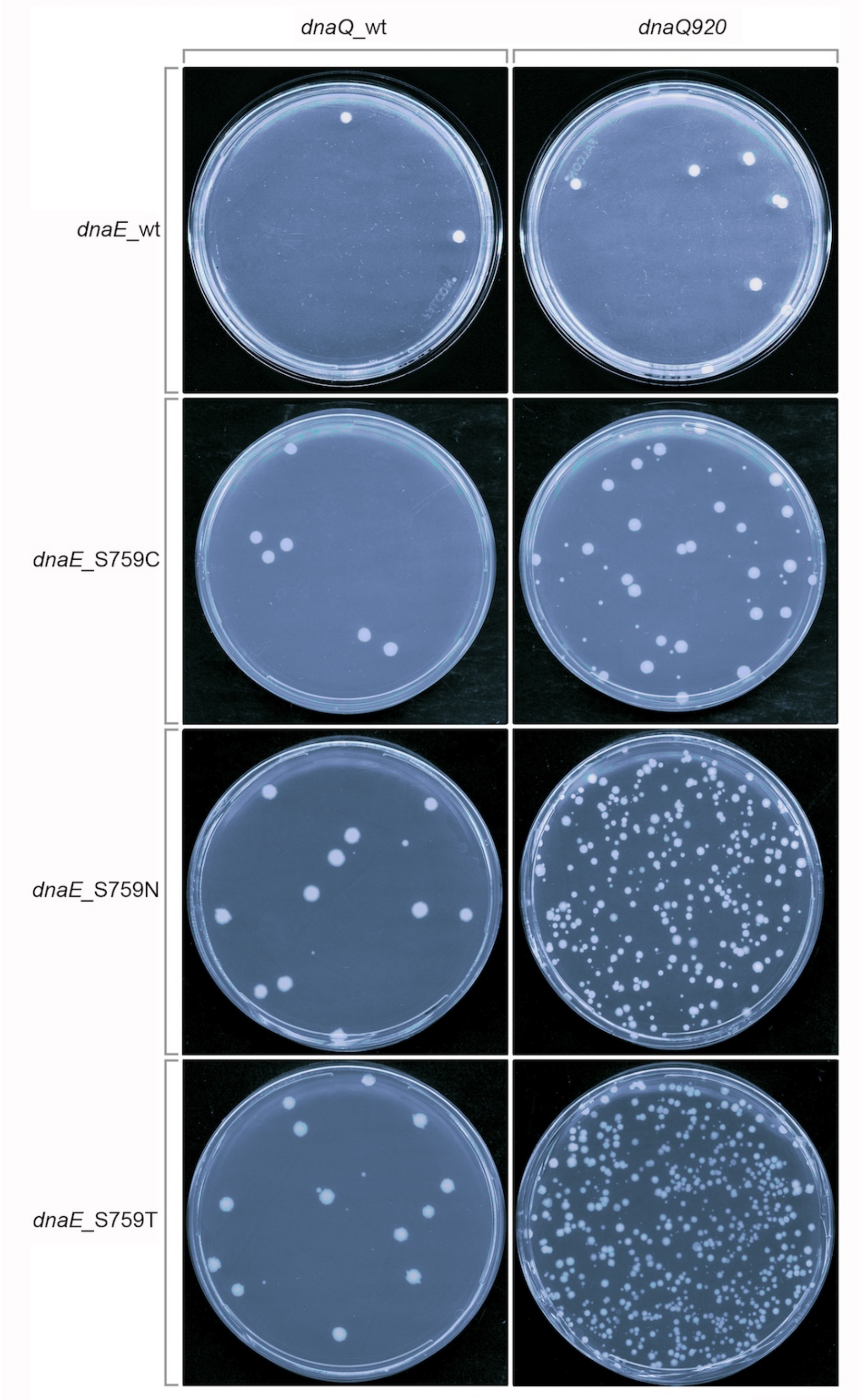

Figure S4. Qualitative plate assay to measure spontaneous mutator activity of wild-type *dnaE* and *dnaE_S759* variants. The images shown are representative of

multiple repeat experiments. Overnight cultures grown in appropriate antibiotics were harvested by centrifugation, then resuspended in SM buffer and 100 μl of the SM solution spread on minimal low histidine plates and incubated at 37 °C for 4 days. His^+ revertants grow up as white colonies on the background "mist" of the His^- parental strain. These experiments reveal that in a *dnaQ*$^+$ strain the three *dnaE* variants are mild-mutators compared to wild-type *dnaE.* Mutator activity increases significantly in *dna920* strains, with strong mutator effects observed with *dnaE*_S759N and *dnaE*_S759T.

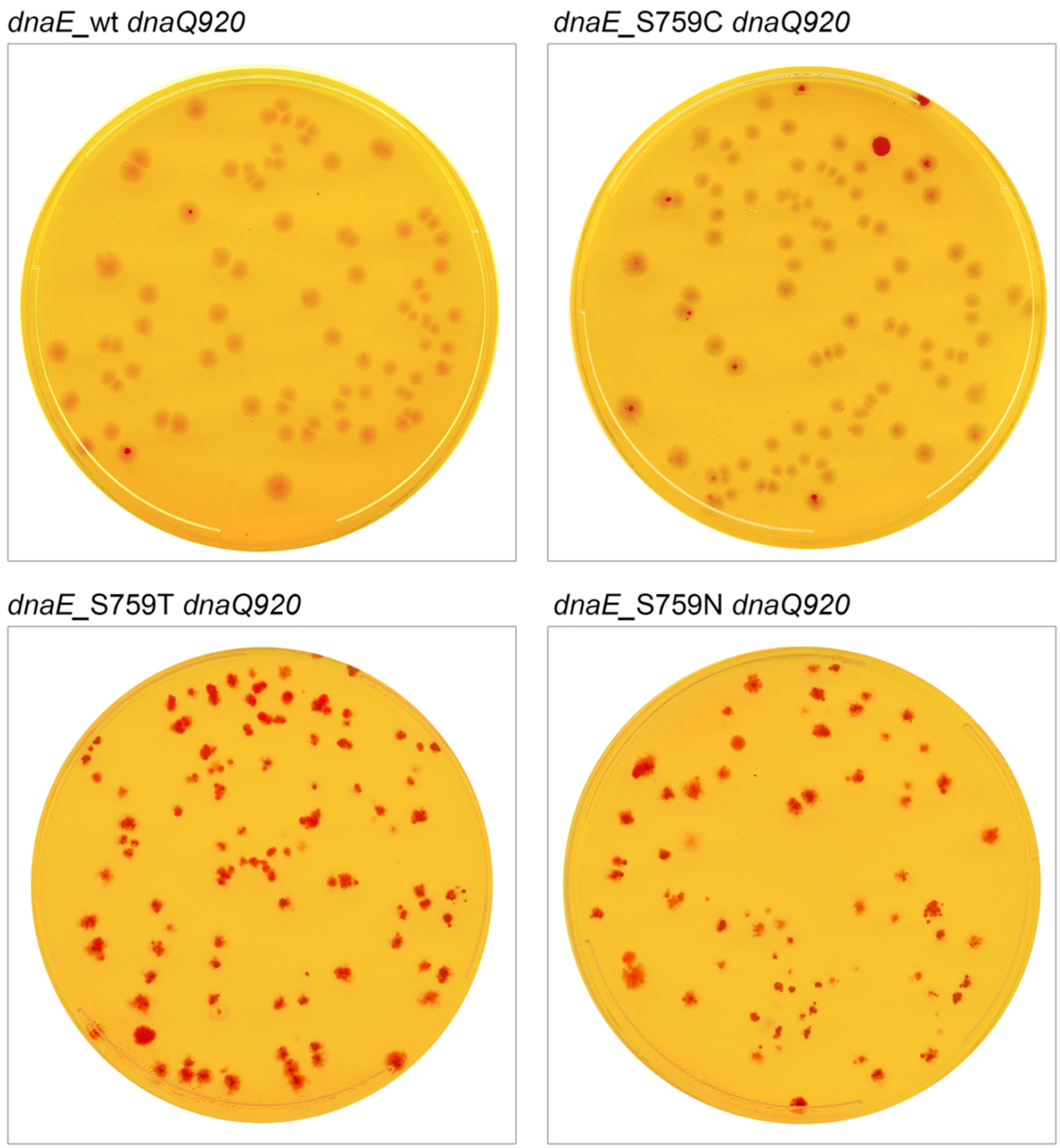

Figure S5. Qualitative papillation assay to measure spontaneous mutator activity of wild-type *dnaE* and S759 variants in *dnaQ920* strains. The images shown are representative of multiple repeat experiments. Overnight cultures grown in appropriate antibiotics were serially diluted up to 10^6-fold in SM buffer. 50 - 100 μl of the diluted

cultures were plated on MacConkey agar base containing 1% galactose and incubated at 37 °C for 8 days. Under these conditions, the Gal$^-$ parental strain appears pink/orange against the background. Bacteria that revert to Gal$^+$ are able to metabolize the galactose in the medium and appear as bright red papillae against the pink/orange strain background. As expected, the wild-type *dnaE dnaQ920* strain (RW1614) exhibited almost no indication of Gal$^+$ papillation. The *dnaE*_S759C *dnaQ920* strain (RW1716) gave a handful of colonies with papillae. In dramatic contrast, virtually all colonies exhibited significant Gal$^+$ papillation with the *dnaE*_S759T or *dnaE*_S759N alleles in the *dnaQ920* background (strains RW1616 and RW1618 respectively).

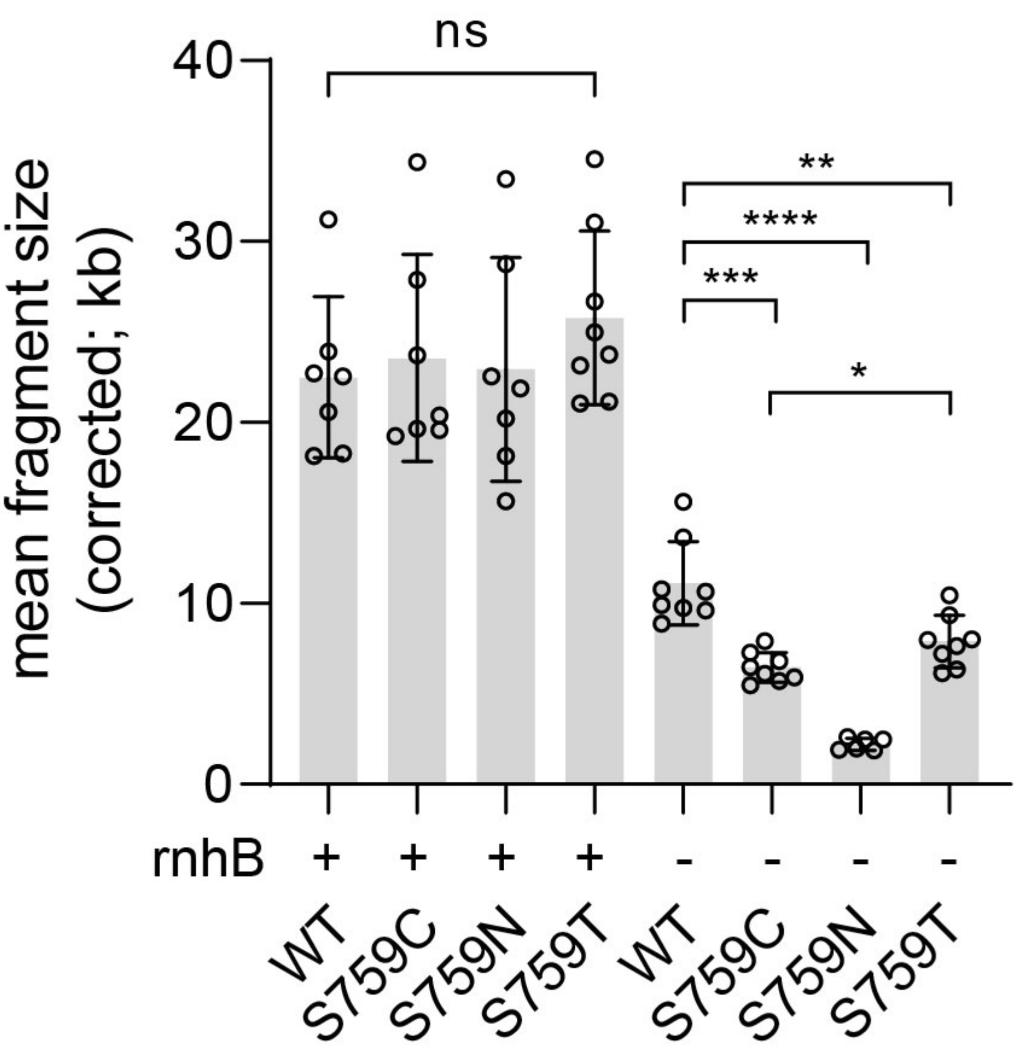

Figure S6. Increased fragmentation of RNase H2-treated genomic DNA from Δ*rnhB*, but not *rnhB*_WT strains expressing *dnaE* mutants. Densitometry plots after alkaline gel electrophoresis of RNase H2-treated genomic DNA were used to calculate the corrected mean fragment size for each sample per gel. This was then used to determine the number of genome-embedded ribonucleotides for the Δ*rnhB* strains relative to the $rnhB^+$ strains (see *Experimental procedures*). Individual data points indicate values from n = 6-8 independent experiments, with bars and error bars indicating mean ± SD. Unpaired 2-sided t-test with Welch's correction; *, p < 0.05; **, p < 0.01; ***, p < 0.001; ****, p < 0.0001; ns, not significant.

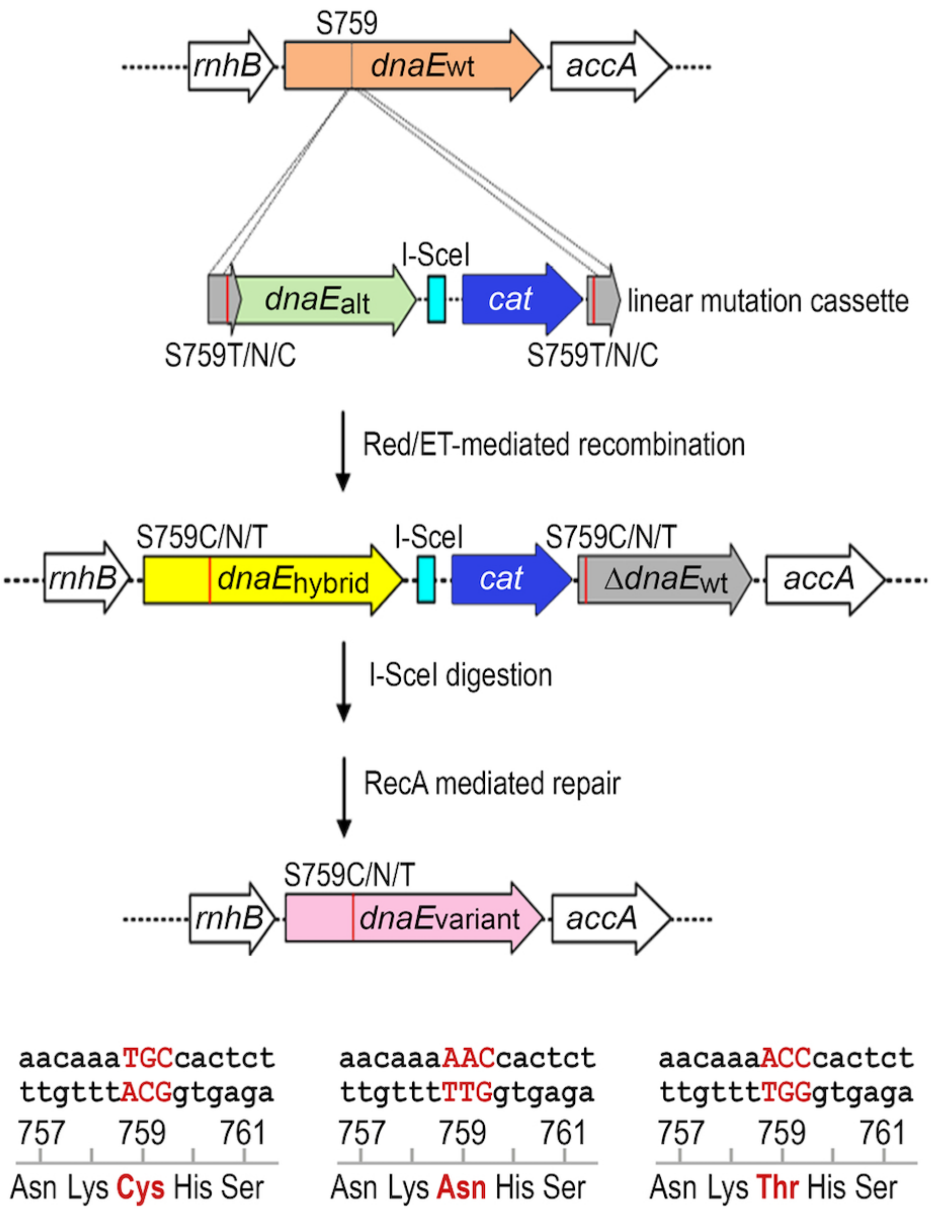

Figure S7. Genome modification strategy to generate the desired point mutations at position S759 in the essential *dnaE* gene. First, a linear mutation cassette was inserted into the chromosomal *dnaE* gene (wt) resulting in a hybrid *dnaE* gene (hybrid) encoding for a fully functional DNA polymerase III α-subunit. The selection marker was removed by a I-*Sce*I restriction and subsequent RecA-mediated repair forming the original endogenous *dnaE* allele including the desired point mutations.

S1 Table. PCR primers

Name	Sequence	Source
rnhB_F55	TTCCGTGAACTGCATCAGCA	Lofstrand
rnhB_R773	GCCATCGATCATCGAGTAGT	Lofstrand
EcdnaE486_F2378	GAC CGC CGA TAT GGA CAA	Lofstrand
EcdnaE486_R2911	GAC ATA ACG CTC AAT CTC TTT	Lofstrand
dnaE_F2059	CTACGGCATTATCCTGTATC	Lofstrand
dnaE_R2557	AAGTAGCCGCCTTTATTACG	Lofstrand
EcdnaQ_F26	ACTGCAATTACACGCCAGAT	Lofstrand
EcdnaQ_R328	CGCTTAAGCAACGAAAACTC	Lofstrand
rpoB1	CACACGGCATCTGGTTGATACG	Lofstrand
rpoF1	TGGCGAAATGGCGGAAAAC	Lofstrand
PCR1-up-S759T	GAACTGGCGATGAAAATCTTCGACCTGGTGGAGAAATTCGC TGGTTACGGATTTAACAAAACCCACTCTGCGGCCTATGCGC TGGTGTCATACCAGACC	BioSpring
PCR2-down-S759T	GGATAGTGCGCTTTCAGCCATAACGTTTGATATGACACCAAA GCATAGGCCGCAGAGTGGGTTTTGTTAAATCCGTTTACGCC CCGCCCTGCCACTCATC	BioSpring
PCR1-up-S759N	GAACTGGCGATGAAAATCTTCGACCTGGTGGAGAAATTCGC TGGTTACGGATTTAACAAAAATCACTCTGCGGCCTATGCGCT GGTGTCATACCAGACC	BioSpring
PCR2-down-S759N	GGATAGTGCGCTTTCAGCCATAACGTTTGATATGACACCAAA GCATAGGCCGCAGAGTGATTTTTGTTAAATCCGTTTACGCCC CGCCCTGCCACTCATC	BioSpring
PCR1-up-S759C	GAACTGGCGATGAAAATCTTCGACCTGGTGGAGAAATTCGC TGGTTACGGATTTAACAAATGCCACTCTGCGGCCTATGCGC TGGTGTCATACCAGACC	BioSpring
PCR2-down-S759C	GGATAGTGCGCTTTCAGCCATAACGTTTGATATGACACCAAA GCATAGGCCGCAGAGTGGCATTTGTTAAATCCGTTTACGCC CCGCCCTGCCACTCATC	BioSpring
cp1	CTGTATCAGGAACAGGTCATG	BioSpring
cp2	GACGATCAAATGCACCTGAC	BioSpring
cp3	GGAAGTAGCCGCCTTTATTAC	BioSpring
cp4	GATCCCGCTGGATGATAAG	BioSpring
cp5	CCATCTAATACCACCTGCTC	BioSpring
cp6	CTGGAGTGAATACCACGAC	BioSpring

Table S2. **Spectrum of spontaneous mutations generated in the *rpoB* gene in MMR-proficient strains.**

	Position	bp change	*dnaE*⁺	S759C	S759N	S759T
	1525	AT→CG	2			
	1532	AT→TA	5			
[	1532	AT→CG	8			
	1532	AT→GC	3		2	3
	1534	AT→GC	6	4	6	10
[	1535	CG→AT	4	9		
	1535	CG→TA	1	1		
	1537	CG→AT	2	1		
	1538	AT→TA	14	10	34	10
[	1538	AT→CG	4	10	2	1
	1538	AT→GC		1	67	53
	1544	AT→GC				1
[	1546	CG→AT		3		3
	1546	CG→TA	1	5	14	42
[	1547	AT→TA	13	50	11	5
	1547	AT→GC	8	6	4	13
	1552	AT→GC	1		1	
	1565	CG→TA			1	
	1576	CG→GC	2	5		
[	1576	CG→AT	1	1		2
	1576	CG→TA			10	5
	1577	AT→TA	22	144	35	24
[	1592	CG→AT		1		
	1592	CG→TA	2	6	74	41
	1596	AT→CG			1	
	1598	AT→GC	1	2		
	1600	CG→AT	1			
	1687	AT→CG	23			
	1691	CG→TA	5		5	18
[	1714	AT→TA	27	112	60	113
	1714	AT→CG	92			
	1715	AT→TA	8			
[	1715	AT→CG	45			
	1715	AT→GC	1			
	Total		302	371	327	344

[a] The data are the number of mutants found for each type of base substitution at a particular position

[b] The numbering system originates from Garibyan *et al*., (DNA Repair. 2003; **2**: 593–608), where the A of the ATG initiation codon is #1. Brackets indicate different types of mutations at the same nucleotide.
